# Signatures and likely sources of the male pregnancy microbiome in wild bay pipefish (*Syngnathus leptorhynchus*)

**DOI:** 10.1186/s42523-025-00476-y

**Published:** 2025-10-27

**Authors:** Elyse M. Barker, Clayton M. Small, Susan Bassham, Emily A. Beck, Mark C. Currey, Hope M. Healey, Bernadette D. Johnson, William A. Cresko, Adam G. Jones

**Affiliations:** 1 Department of Biological Sciences, University of Idaho, Moscow, ID USA; 2 Department of Data Science, University of Oregon, Eugene, Oregon, USA; 3 Institute of Ecology and Evolution, University of Oregon, Eugene, Oregon, USA; 4 Department of Molecular Biosciences, University of Kansas, Lawrence, Kansas, USA; 5 Knight Campus for Accelerating Scientific Impact, University of Oregon, Eugene, Oregon, USA; 6 Biodiversity Research Centre, University of British Columbia, British Columbia, Canada

**Keywords:** Host-microbe interactions, Evolutionary novelty, Male pregnancy, Syngnathidae, Pipefish

## Abstract

**Background:**

Understanding the origin and structure of microbiomes and their associations with ecologically significant host traits is essential for understanding the evolution of host-microbe interactions. These interactions support a wide range of physiological processes important for development, survival, and reproduction. Syngnathid fishes (seahorses, pipefish, and seadragons) represent a compelling system for investigating host-microbiome interactions due to their unique evolution of male pregnancy. Males harbor a fitness-critical brood pouch that provides embryos with protection, osmoregulation, and nutrient exchange through a placenta-like structure, all while requiring the male to modulate his immune system to accommodate developing offspring. These features create a tightly regulated internal environment where microbial interactions could be especially influential to supporting a successful pregnancy. While we have some understanding of the physiological and genetic factors underlying brood pouch development and maintenance, the role of the microbiome and host-microbe interactions in male pregnancy has remained underexplored across the broader diversity of Syngnathidae species and geographic regions. To investigate this relationship further, and for the first time sampling microbiota from a wild syngnathid population, we characterized microbiomes of the bay pipefish (*Syngnathus leptorhynchus*) using high-throughput 16S rRNA gene sequencing. We quantified microbial community diversity and composition across the brood pouch, embryos, ovaries, gills, intestines, and outer skin body tissues, focusing on sex-specific (brood pouch, embryo, ovary) and sex-shared (gill, intestine, skin) tissues, and variation in pregnancy stage (non-pregnant, early, mid, and late pregnancy).

**Results:**

We found that the male brood pouch microbiome was distinct from all other body sites (ovaries, embryos, gills, intestines, and outer skin) in community composition, and in that it exhibited the highest richness and phylogenetic diversity of microbes of any site on average, possibly supporting a specialized environment for embryonic development. Moreover, we found that microbial diversity was lower in non-pregnant brood pouches compared to each pregnancy stage (non-pregnant, early, mid, and late pregnancy) but found no significant differences among the pregnancy stages. Female ovaries had the lowest microbial richness and phylogenetic diversity compared to nonpregnant brood pouches, pregnant brood pouches, and embryos. Source tracking analysis using fast expectation-maximization for microbial source tracking (FEAST) indicated that the male outer skin serves as a significant microbial source for both the pregnant brood pouch and developing embryos, establishing a strong paternal influence on the offsprings’ microbial communities. Overall, we identified Proteobacteria, Bacteroidota, Cyanobacteria, Planctomycetota, and Actinobacteriota as the dominant phyla spanning all surveyed bay pipefish tissue sites, consistent with previous teleost fish microbiome studies. Analysis of core microbiome and indicator species further revealed that sequences classified as *Methylotenera_A_557637* (two species), *GCA-2862085* sp., *Yoonia_491068* sp., *Pla163 sp007750655*, and *Roseibacillus_*B sp. show relatively high abundance and specificity with respect to the male brood pouch, suggesting that these taxa may have functional connections to the biology of male pregnancy.

**Conclusions:**

These findings reveal insights into the microbial ecology of a unique reproductive system in its natural environment, highlighting the paternal microbiome’s potential functional role in shaping the developing offspring. Our results also indicate a likely influence of both environmental and host-specific factors in shaping the bay pipefish microbiome, but there is need for future research on the functional implications of these microbial communities, especially in the brood pouch during pregnancy, and with respect to offspring viability and fitness.

**Supplementary Information:**

The online version contains supplementary material available at 10.1186/s42523-025-00476-y.

## Background

Research on animal microbiomes has repeatedly shown that microbial communities are highly diverse and play an essential role in the development, overall health, and metabolism of the host [1–3]. Diversity and structure of taxa within the microbiome often help determine animal growth, health, and survival outcomes, a fundamental insight gleaned from host-microbe research in teleost (i.e. “bony”) fishes, a group heavily studied for their taxonomic, phenotypic, and ecological diversity [4]. While many of these fish studies focus on the gut microbiome, the reproductive microbiome is still poorly understood, especially in species with specialized reproductive structures. Furthermore, much of the teleost microbiome literature focuses on model organisms, such as zebrafish and three-spined stickleback [5–7], or commercially important aquaculture fish species like tilapia and salmon [8], leaving gaps in our understanding of microbial diversity in a broader range of less-studied fish species. Although there are advantages to studying host-microbiome relationships using model organisms, there is a fundamental gap in understanding host-microbe interactions in fishes with novel traits, which may reveal how microbial communities facilitate host adaptation to extreme or unique ecological conditions, perhaps through host filtering of microorganisms that exploit specific host resources or can perform biochemical tasks that the host cannot. Here, the family Syngnathidae (pipefishes, seahorses, and seadragons) serves as a compelling example of a taxon with a unique novel reproductive trait, male pregnancy, that is not seen in any other known species. During reproduction, the female passes her nutrient-rich eggs to the male’s pouch, and he fertilizes them as they are being transferred [9]. The male harbors a highly vascularized brood pouch that incubates the embryos. This male brood pouch varies in morphological complexity across syngnathids, ranging from a completely open pouch with externally glued eggs to a fully enclosed pouch with a placenta that provides oxygen, nutrition and removes waste [10, 11]. The novel reproductive strategies of syngnathids offer a compelling model for exploring host-microbe interactions, including mechanisms of vertical microbial transmission, with recent studies revealing striking patterns in the pipefish microbiome. Studies mainly using the European broadnosed pipefish, *Syngnathus typhle*, have revealed that while maternal microbes are foundational for establishing the juvenile gut microbiome, paternal microbial transmission significantly shapes the external microbiome and can even confer adaptive advantages such as improved offspring survival [12, 13]. In addition, temporal changes in microbial communities have been observed across the course of pregnancy, with late-stage pregnancy displaying distinct microbial compositions compared to earlier stages. These shifts may result from changing microbial sources, including the introduction of environmental microbes through increased brood pouch permeability, potentially preparing embryos for their future microbial environment [12, 14]. Other studies have shown that maternal and paternal gonads, along with paternal brood pouches, harbor distinct microbial communities, suggesting a sex-specific influence on embryo development [14]. Additionally, experimental studies have demonstrated that paternal microbiome manipulation using spike-in bacterial treatments of bacterial strains identified as potentially involved in vertical paternal transmission significantly impacted offspring microbiome establishment and development [13]. Despite these compelling findings in *S. typhle*, microbiome studies are limited across the diversity of *Syngnathus* species, particularly in fish sampled directly from the wild. Instead, many Syngnathidae microbiome studies source their fish from aquaculture facilities or use experimental designs requiring captive housing [14–17]. While experimental manipulations are valuable for testing hypotheses that require controlled conditions like disentangling parental transmission, non-wild sampling has been found to alter the microbiome diversity in several vertebrate species, potentially leading to conclusions that deviate from patterns observed in natural conditions [3, 18–22]. No published study has yet characterized the microbiome of any *Syngnathus* species directly from their natural habitats. Furthermore, there is a clear need to sample a greater diversity of body sites in syngnathid microbiome studies, using host species from diverse regions and habitats, to more fully understand microbial diversity across this family’s widespread global distribution, which encompasses all ocean basins except the polar regions [23]. Broader body site sampling can reveal tissue-specific microbial communities, uncover patterns of microbial specialization or overlap across sites, and provide insight into how different body regions contribute to overall host-microbe interactions.

Here, we focus on the bay pipefish, *Syngnathus leptorhynchus*, a marine species inhabiting the Eastern Pacific Ocean from Southern California to Alaska, mainly found in vegetation such as eelgrass beds [24]. The bay pipefish is polygynous, with males mating with multiple females [25]. It lives one to two years and can produce up to twelve broods of hundreds of offspring [26]. The *S. leptorhynchus* brood pouch morphology is characterized by eggs embedded in a spongy brooding matrix and enclosed from the external environment by skin folds that adhere to each other to form a sealed pouch until parturition [27]. As there are limited studies on Syngnathidae microbiomes, *S. leptorhynchus* has not been investigated in any capacity with respect to its microbiome and was chosen to provide complementary data that expands upon previous Syngnathidae microbiome research. Characterizing a North American syngnathid offers an opportunity to compare microbiomes across species and regions, revealing how geographic distance and species-specific traits may influence microbial communities. Expanding research beyond a few focal species is essential for capturing the full range of host–microbe interactions within this diverse and globally distributed family.

In this study, we used high-throughput 16S rRNA sequencing to characterize the microbiome of the wild-caught bay pipefish (*Syngnathus leptorhynchus*) across six body sites, spanning both males and females. This sampling included male brooding tissue across pregnancy stages, which allowed us to explore potential host-microbe interactions that support male pregnancy and embryonic development. Our analyses aimed to characterize the wild microbiome of *S. leptorhynchus* by (1) identifying and comparing the microbial composition and diversity of different body sites, sexes, and pregnancy stages, (2) testing for brood pouch-specific effects on the composition of the overall microbiome using core and indicator species analysis, and (3) investigating the potential microbial origins of the pregnant brood pouch and embryo microbiomes using source tracking analysis. Our focus on the microbiome of male brooding tissues in pipefish from a wild population provided an opportunity to examine microbial roles in male pregnancy, leading to a deeper understanding of how host-microbiome interactions drive unique reproductive adaptations in natural habitats.

## Results

### Overall bay pipefish microbiome characterization

**Fig. 1:**
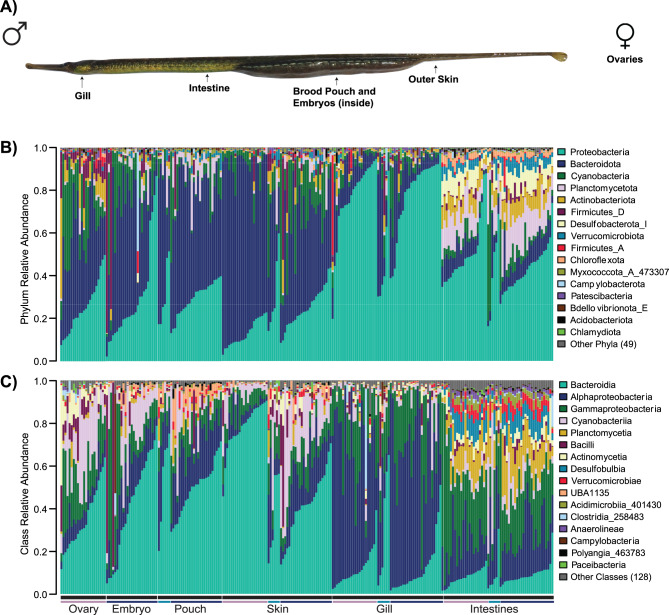
Variation in the composition of assigned microbial taxonomy across bay pipefish body sites. (**A**) bay pipefish body sites sampled, as shown on an image of a pregnant male. For females (not pictured), there are no brood pouch or embryo samples, and we included ovary samples. (**B**) relative abundance bar plot by phylum. (**C**) relative abundance bar plot by class. Bar color above body sites indicates non-pregnant males (light blue), pregnant males (dark blue) and female (violet) individuals

We characterized and quantified the bay pipefish microbiota using high-throughput 16S rRNA sequencing on replicate diverse tissues that included 31 brood pouch samples (6 non-pregnant, 13 early pregnancy, 8 mid pregnancy, 4 late pregnancy), 25 embryo samples corresponding to pregnancy stage (13 early, 8 mid, 4 late development), 52 gill samples, 54 intestine samples, 20 ovary samples, and 53 skin samples (Fig. 1A). There were a total of 22,523 amplicon sequence variants (ASVs) and 805,220,579 reads, with a mean of 3,246,857 reads per sample. The samples exhibited a wide range of read counts, from a minimum of 4,404 to a maximum of 17,561,384. After filtering the ASV table as reported in the methods, we retained 235 samples with 9,633 features and 613,187,701 reads. The overall bay pipefish microbiome consisted of 65 bacterial phyla, with the 13 most abundant phyla accounting for 99% of the total relative abundance. These dominant phyla included Proteobacteria (40.8%), Bacteroidota (31.8%), Cyanobacteria (9.1%), Planctomycetota (5.0%), Actinobacteriota (3.4%), Firmicutes_D (2.9%), Desulfobacterota_I (1.9%), Verrucomicrobiota (1.6%), Firmicutes_A (0.6%), Chloroflexota (0.6%), Myxococcota_A_473307 (0.4%), Patescibacteria (0.4%), and Campylobacterota (0.3%) (Fig. 1B). The most abundant phyla, (Proteobacteria, Cyanobacteria, and Bacteroidota), were present in all body site types. Several ASVs were ubiquitously present across all host samples: *Unclassified Flavobacteriaceae*, *SIO2C1 sp010672925*, *Unclassified Rhodobacteraceae*, and *Unclassified Burkholderiales_592524*. Other phyla were exclusive to a single site, including Firmicutes_E and Firmicutes_B_370525 in the intestines and Delongbacteria in the brood pouches. Some phyla were shared among a few body sites: Atribacterota, Calditrichota, and Desulfobacterota_E were present only in brood pouches and gills; Fermentibacterota, Desulfobacterota_D, and Firmicutes_B_370543 were present only in brood pouches and skin sites; and Moduliflexota was present only in brood pouches and intestines (Supplemental Fig. 1). Within these phyla, there were a total of 144 classes, the most abundant of these being Bacteroidia (31.8%), Alphaproteobacteria (23.6%), Gammaproteobacteria (17.2%), Cyanobacteriia (9.1%), Planctomycetia (3.8%), Bacilli (2.9%), Actinomycetia (2.3%), Desulfobulbia (1.8%), Verrucomicrobiae (1.6%), and UBA1135 (1.2%) (Fig. 1C).

### Microbiome diversity differs across body sites, highlighting a distinct brood pouch microbial community

**Fig. 2:**
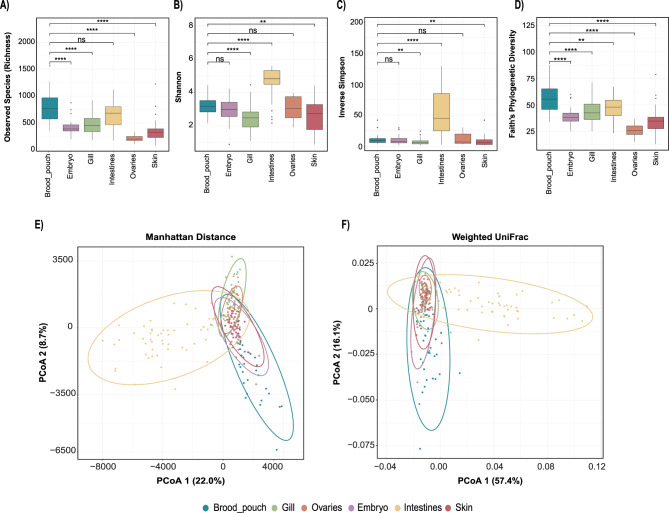
Alpha and beta diversity of ASVs between and among bay pipefish body sites. Alpha diversity metrics by (**A**) observed species richness, (**B**) shannon index, (**C**) inverse Simpson index, and (**D**) Faith’s phylogenetic diversity. Two-dimensional principal coordinates analysis (PCoA) of microbial communities across all body sites, using (**E**) Manhattan distance and (**F**) weighted UniFrac metrics with 95% confidence ellipses. Pairwise test results are marked by horizontal lines connecting group bars for select group pairs, and statistically significant differences are denoted using asterisk notation (*p* > 0.05 ns, *p* ≤ 0.05*, *p* ≤ 0.01**, *p* ≤ 0.001***, *p* ≤ 0.0001****). For select pairwise test results not shown, see table S2

We compared alpha diversity (within-sample diversity) among body sites using richness (number of ASVs), Faith’s phylogenetic diversity (Faith’s PD), Shannon, and Inverse Simpson indices (Fig. 2A–D). The male brood pouch had the highest richness, on average and was significantly different from embryo, ovary, gill, and skin sites (Wilcoxon Test corrected p-values < 0.001, respectively; Fig. 2A, Table S2), but was not significantly different from the intestines (Wilcoxon Test corrected p-value = 0.190; Fig. 2A, Table S2). A similar trend was observed in Faith’s PD index, which measures the diversity based on the evolutionary relationships among microbial species. The brood pouch had the highest phylogenetic diversity, significantly differing from all other body sites (Wilcoxon Test corrected p-values < 0.001; Fig. 2D). Shannon and Inverse Simpson diversity metrics (which quantify richness and evenness to different extents) both showed the same pattern of among-site differences, in which brood pouch diversity was significantly higher than gill and skin diversity, but lower than gut samples, on average (Wilcoxon Test corrected p-values < 0.01; Fig. 2B–C). The brood pouch microbiome exhibited high species richness and Faith’s PD, but lower Shannon and Inverse Simpson diversity compared to other sites. This suggests a diverse community with many taxa present, but low evenness, where a few species dominate and most are present at low abundance. The intestine samples demonstrated the highest microbial diversity according to the Shannon Index and Inverse Simpson Index, despite not having the highest species richness or phylogenetic diversity (Fig. 2A–D). This indicates a diverse and relatively even microbial community at the ASV level within the intestines.

To assess beta diversity (between-sample diversity) we applied a measure of abundance-based community dissimilarity (Manhattan distance) at the ASV level. We used PERMANOVA to test whether factors in our study (namely body site) explained variation in microbial community. Body site explained the most variation in Manhattan Distance (PERMANOVA: = 0.245, F = 14.825, *p* ≤ 0.001), followed by pregnancy stage (PERMANOVA: R^2^ = 0.039, F = 1.956, *p* ≤ 0.001), sex (PERMANOVA: R^2^ = 0.029, F = 7.074, *p* ≤ 0.001), and lastly pregnancy status (pregnant versus non-pregnant) (PERMANOVA: R^2^ = 0.016, F = 2.447, *p* = 0.007) (Table S3). All p-values were significant, indicating that body site, pregnancy stage, sex, and pregnancy status each have a significant influence on overall microbiome dissimilarity. PERMANOVA results showed that the microbial communities are significantly distinct between the different body sites (R^2^ = 0.245, F = 14.825, *p* ≤ 0.001). Additionally, PERMDISP analysis showed significant differences in the dispersion of microbial communities across these sites (F = 48.809, *p* ≤ 0.001), suggesting that there are differences at the level of group centroids, but also differences in the degree of beta diversity (variation among fish) across body sites. Specifically, brood pouch and intestine body sites showed high levels of dispersion relative to other sites. Pairwise PERMANOVA (Table S3) showed that the brood pouch sites were significantly different (*p* ≤ 0.001) from all the other body sites. This is visually confirmed in the PcoA plot (Fig. 2E), where the brood pouch sites cluster away from the other sites. The intestines form another distinct cluster on the PcoA plot (Fig. 2E). Although there was significant overlap between the ovaries, embryos, gill, and skin in the PcoA plot, the body sites were all significantly different from each other in the full-dimensional community space, according to PERMANOVA (Table S3).

We observed similar trends when analyses were based on phylogenetic community dissimilarity using weighted UniFrac across all body sites. Body site explained the most variation in weighted UniFrac (PERMANOVA: R^2^ = 0.474, F = 41.215, *p* ≤ 0.001), followed by pregnancy stage (PERMANANOVA: R^2^ = 0.033, F = 1.651, *p* = 0.092), sex (PERMANOVA: R^2^ = 0.029, F = 6.885, *p* = 0.003) and pregnancy status (PERMANOVA: R^2^ = 0.017, F = 2.548, *p* = 0.043) (Table S3). However, pregnancy stage was not significant (*p* = 0.092), which indicates that both abundance-based and phylogenetic distance among samples is not explained well by differences in pregnancy stage. The analysis of beta diversity using weighted UniFrac revealed significant differences in microbial community abundance and phylogenetic relatedness across all body sites (Table S3, PERMANOVA: *p* < 0.05). However, PERMDISP analysis also revealed significant differences in the dispersion of microbial communities within each body site (Table S3, PERMDISP: *p* < 0.05). This implies that the differences inferred from the PERMANOVA are influenced by both the distinct composition of microbial communities and their variability across fish within each body site. The weighted UniFrac PcoA plot (Fig. 2F) revealed distinct clustering of the brood pouch and intestines, showing minimal overlap with other body sites and mirroring the clustering pattern observed with the Manhattan distance. Specifically, the brood pouch and intestines exhibit greater variability in microbial community composition, as indicated by the wider spread of points, compared to the more tightly clustered gills, ovaries, outer skin, and embryos. While PERMANOVA indicates significant differences between all body sites for weighted UniFrac (Table S3), the PcoA plot reveals overlap among the embryo, ovary, outer skin, and gill samples. This suggests that, despite the overall significant differences, these body sites are more similar and share more phylogenetically related taxa than the brood pouch and intestines.

### Core microbiomes of bay pipefish body sites include specialist and generalist taxa

In our analysis of microbiome composition, we identified the ‘core’ microbiome for each body site. We defined the core microbiome as the 25 most abundant ASVs present in at least 50% of the samples from each respective body site (Table S4). While environmental samples were not included, the high abundance and prevalence of these taxa within specific tissues support their classification as core, despite the possibility that some may be environmentally derived.

The brood pouch, embryo, gill, intestines, and ovaries each had unique core ASVs exclusive to their particular sites (6, 1, 9, 12, and 6 ASVs, respectively). However, the skin had no unique core ASVs and instead shared its 15 core taxa with other body sites. The core ASVs that are present in every body site are *Croceitalea litorea*, *SIO2C1 sp010672925*, and *Unclassified Rhodobacteraceae*. These 3 bay pipefish core taxa are consistently found in almost every sample and exhibit some of the highest relative abundances, regardless of the body site.

### Core microbiome and indicator species analyses reveal key brood pouch taxa

The brood pouch core microbiome was dominated by the phyla Bacteroidota, Planctomycetota, and Proteobacteria, with key families including *Flavobacteriaceae*, *Rhodobacteraceae*, and *Methylophilaceae*. Some of the most abundant agglomerated ASVs in the brood pouch, including *Croceitalea litorea*, *Spongiimicrobium salis*, and *Croceitalea sp.*, were present in over half of the brood pouch samples, regardless of pregnancy status. The core taxa unique to the brood pouch samples, along with their mean relative abundances, were *Methylotenera_A_557637 oryzisoli* (1.6%), *GCA-2862085 sp.* (1.4%), *Yoonia_491068 sp.* (1%), *Pla163 sp007750655* (0.84%), *Methylotenera_A_557637 mobilis* (0.73%), and *Roseibacillus_B sp.* (0.70%) (Table S4). The brood pouch and embryo shared the core species *Croceitalea sp.*, while the brood pouch and intestines shared two core species, *Fuserstia sp*. and *Mariniblastus sp011087765*. The brood pouch, embryo, ovaries, and skin shared *Spongiivirga citrea* and *Arenicella sp.* as core ASVs.

A total of 43 agglomerated ASVs were present in 100% of brood pouch samples, 16 of which met the criteria for inclusion in the brood pouch core microbiome based on their consistent presence and high abundance. These core taxa included: *Unclassified Flavobacteriaceae*, *Spongiivirga citrea*, *Spongiimicrobium salis, Croceitalea litorea, Portibacter lacus, GCA-2862085 sp., Fuerstia sp., Mariniblastus sp011087765, Roseibacillus_B sp., SIO2C1 sp010672925, Litoreibacter sp., Unclassified Rhizobiaceae_A_499470, Unclassified Rhodobacteraceae, Sulfitobacter_E_490551 sp., Arenicella sp.,* and *Methylotenera_A_557637 oryzisoli.* Of the remaining 27 agglomerated ASVs present in all brood pouch samples, the most abundant were *Unclassified Gammaproteobacteria*, *Unclassified GCA-002705445, Unclassified Sphingomonadaceae,* and *Cocleimonas flava* which were all under 0.6% relative abundance. Thirty-two non-core agglomerated ASVs were found exclusively in the brood pouch sites and not in any other body sites. The most abundant among these were *Leucobacter viscericola* (0.0013%, 6.5% prevalence), *Unclassified UBA9160* (0.001%, 22.6% prevalence), *Kriegella sp.* (0.00072%, 12.9% prevalence), and *Jejudonia soesokkakensis* (0.00023%, 19.4% prevalence). However, despite being unique to the brood pouch, their prevalence and abundances were low, so they are neither part of the “core” microbiome nor among the most abundant taxa, possibly suggesting that they represent very rare male pregnancy-specific taxa not adequately sampled given the sequencing depth of our study.

**Fig. 3:**
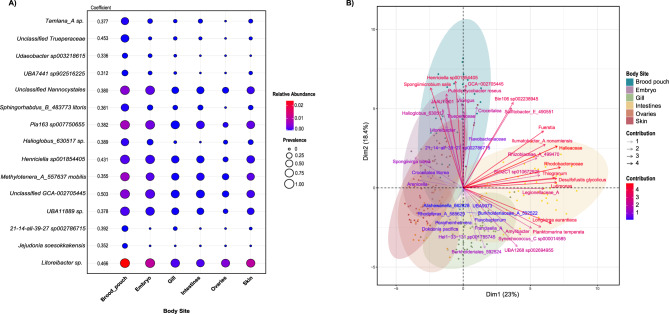
(**A**) indicator species analysis identifies ASVs significantly associated with the brood pouch (*p* ≤ 0.001). The plot displays only the taxa considered indicator species for the brood pouch but includes all body sites to show differences in mean relative abundance and prevalence. Indicator values, ranging from zero to one, denote site fidelity, with higher values indicating stronger association with the brood pouch body site. Circle size corresponds to prevalence, while circle color corresponds to relative abundance. (**B**) PCA biplot illustrating the top 5 highly associated indicator species from each body site

To further identify species significantly associated with the brood pouch tissue, we performed indicator species analysis. Each microbial taxon identified as an indicator species is assigned a coefficient that reflects its strength of association with a particular body site (fidelity). Higher coefficients indicate taxa that are consistently found in their associated sites, making them reliable indicators for those environments. In this analysis, 275 species were identified as indicator species for the different body sites with a significance level (alpha) of 0.001. There were 15 indicator species in the brood pouch, one in both the embryo and skin, 35 in the gill, 181 in the intestines, and 22 in the ovaries (figshare dataset: https://figshare.com/s/37edb68a3c039170265a). Figure 3 shows the 15 ASVs identified as indicator species for the brood pouch, including their relative abundance and prevalence across body sites, along with a principal component analysis (PCA) biplot highlighting those contributing most to site-level differences. This comparison highlights how these taxa differ between the brood pouch and the other body sites. The ASVs with the highest coefficients, and therefore those most associated with the brood pouch, were *Unclassified GCA-002705445* (0.503), *Litoreibacter sp.* (0.466), and *Unclassified Trueperaceae* (0.453). *Litoreibacter sp*. was highly abundant and prevalent across all body sites but was only considered an indicator species for the brood pouch given its consistently high relative abundance across many individual samples. Meanwhile, taxa like *Unclassified GCA-002705445* and *Unclassified Nannocystales* showed high prevalence across sites but were most abundant in the brood pouch. *Jejudonia soesokkakensis*, though somewhat prevalent in the brood pouch at a low abundance, was not present in other body sites. *Putridiphycobacter roseus* was the only indicator species identified in embryos and was not identified as an indicator species in either the maternal (ovaries) or paternal (brood pouch) body sites.

There were also species whose patterns of abundance were more associated with a combination of body sites. For example, the species *Spongiivirga citrea* showed a positive association with the brood pouch, embryo, ovaries, and skin, with a coefficient of 0.322. This suggests that *Spongiivirga citrea* is more frequently found in these sites compared to others. Similarly, *SIO2C1 sp010672925* had a positive association with the ovaries, embryo, and skin, with a coefficient value of 0.402, indicating that this ASV is more common in these sites than in other body sites. Only 3 ASVs were present in both core microbiome and indicator species analyses for the brood pouch: *Litoreibacter sp.* (0.466), *Pla163 sp007750655* (0.382), and *Methylotenera_A_557637 mobilis* (0.355). Their prevalence and abundance in the brood pouch suggest that these species may play a significant role in male pregnancy.

### Pregnancy status contributes to variation in the bay pipefish microbiome

**Fig. 4:**
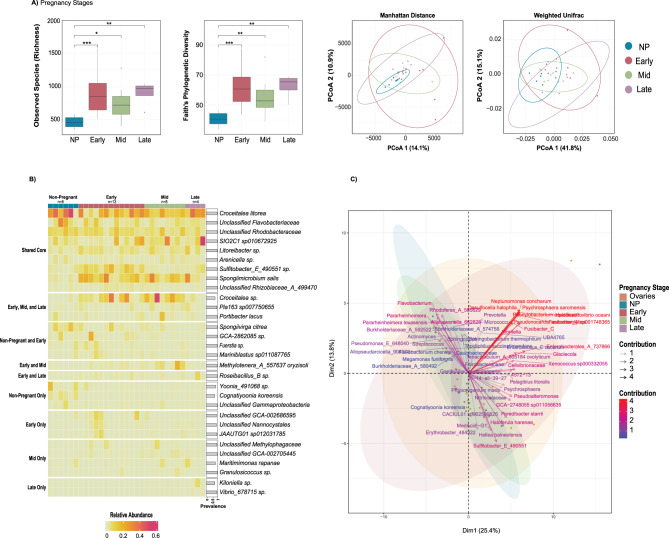
(**A**) Richness, Faith’s PD (alpha diversity), Manhattan distance, and weighted UniFrac metrics (beta diversity) of ASVs were analyzed for brood pouch samples by pregnancy stage (non-pregnant [NP], early, mid, late). Pairwise test results are marked by horizontal lines connecting group bars for select group pairs, and statistically significant differences are denoted using asterisk notation (*p* > 0.05 ns, *p* ≤ 0.05*, *p* ≤ 0.01**, *p* ≤ 0.001***, *p* ≤ 0.0001****). For select pairwise test results not shown, see table S2. Two-dimensional principal coordinates analysis (PcoA) of microbial communities was conducted exclusively for brood pouch sites with 95% confidence ellipses. (**B**) Heatmap of the relative abundance (main cells) and prevalence (grey bars on the right) of the core microbiome in the brood pouch body sites across pregnancy stages. ASVs are shown at the lowest taxonomic level. The top block highlights ASVs that met core criteria (see methods) for all pregnancy stages, with subsequent blocks showing ASVs that met core criteria for a combination of various pregnancy stages, and lastly, ASVs that met core criteria for individual pregnancy stages only. (**C**) PCA biplot of indicator species for pregnancy stages (brood pouch samples only) and ovaries

We used alpha diversity, again measured by richness, Shannon, Inverse Simpson, and Faith’s PD indices to assess pairwise microbial diversity differences between pregnancy statuses (non, early, mid, and late pregnancy) (Fig. 4A) and to compare distributions of non-pregnant males and all pregnant males as a single group regardless of pregnancy stage (Supplementary Fig. 3A). No significant differences were observed between pregnancy stage pairings (early, mid, and late pregnancy) (Table S2 and S3). Observed richness and Faith’s PD represented the only statistically significant alpha diversity differences between non-pregnant brood pouches and each of the three pregnancy stages (early, mid, and late) (Fig. 4A), with non-pregnant brood pouches exhibiting lower richness and phylogenetic diversity in all cases (Table S2).

With respect to overall community similarity (i.e. beta diversity), we noted some clustering of microbial communities corresponding to different pregnancy stages (Fig. 4A), primarily in terms of lower dispersion among non-pregnant brood pouches relative to the other status groups, and for the analysis based on Manhattan distances. The limited sample size and the absence of precise days post-fertilization cutoffs may have increased variability within pregnancy stages, potentially reducing the clarity of shifts in diversity and community composition across stages. The overlap among these ellipses, particularly between early pregnancy and all other stages show that they are not entirely distinct, and there is substantial shared microbial diversity. This is further supported by PERMANOVA, which shows that significant differences in microbiome composition exist only between non-pregnant brood pouches and the other stages (*p* ≤ 0.05), with no significant differences observed between the early, mid, and late pregnancy stages (Table S3). The brood pouch microbiome group dispersion differs between some pregnancy stage comparisons as indicated by PERMDISP (Table S3), specifically between non-pregnant and early stages, as well as non-pregnant and late stages. This suggests that the observed differences among these stages may be influenced by variation in the within-group variability of microbial communities, in addition to differences in community composition between groups. The weighted UniFrac PcoA ordination suggested substantial overlap in community composition among the different pregnancy statuses (Fig. 4A). Despite this overlap, and similar to the Manhattan distance-based tests, PERMANOVA indicated significant differences between non-pregnant status and each pregnancy stage (early, mid, and late) (*p* ≤ 0.05), with no significant differences found among the early, mid, and late pregnancy stages (Table S3).

### Core microbiomes and indicator species reflect taxonomic consistency and fidelity with changes in pregnancy status

Overall, brood pouch sites harbored a total of 58 phyla. Pregnant and non-pregnant brood pouches shared 29 phyla, with non-pregnant brood pouches having one unique phylum (Armatimonadota) and pregnant brood pouches having 28 unique phyla (Supplementary Fig. 2). We inferred core microbiomes by pregnancy status (non, early, mid, and late pregnancy) for the brood pouch site (Fig. 4B) to investigate whether certain taxa are consistently present and abundant across different pregnancy stages, potentially indicating their role in maintaining microbial stability throughout male pregnancy and supporting specific reproductive functions at each stage. There were 9 core ASVs shared across all pregnancy stages (Fig. 4B). While these shared core microbes were found in all brood pouch samples for all pregnancy statuses, their abundance fluctuated throughout the course of pregnancy. *Croceitalea litorea* had the highest overall abundance and was a shared core taxon across all pregnancy statuses (28.6%, 16.3%, 13.3%, and 24.9%, respectively). Some shared core species, including *Unclassified Flavobacteriaceae*, *Croceitalea litorea*, and *Unclassified Rhodobacteraceae* were the most abundant in non-pregnant brood pouches (10.2%, 28.6%, and 7.6%, respectively) and decreased in abundance as the pregnancy progressed. Each pregnancy status harbored some unique core ASVs that could indicate functional importance to those specific stages during embryo development. Notably, late pregnancy contained 2 unique ASVs, with *Kiloniella sp.* being the most abundant at 1.5% and present in all samples, followed by *Vibrio_678715 sp.* with 0.4% abundance and occurrence in all samples. Three core species were shared across early, mid, and late stages; 4 between non-pregnant and early stages; and 1 between early and mid-stages, and early and late stages.

We also performed indicator species analyses for brood pouch samples (Fig. 4C, Fig. S4) to identify taxa associated with the specific stages of pregnancy. There were 8 indicator species for non-pregnant brood pouches, with *Cognatiyoonia koreensis* (0.746) and *Erythrobacter_484322 sp.* (0.635) showing the highest coefficients and therefore exhibiting high site fidelity within the non-pregnant brood pouch. *Cognatiyoonia koreensis*, was unique to the non-pregnant core microbiome and had the highest abundance in non-pregnant brood pouches compared to early, mid, and late stages (2.3% vs. 0.1%, 0.1%, and 0.3%, respectively). Early pregnancy had just 1 indicator species, *Sulfitobacter_E_490551 sp.* (0.699), which was also part of the core microbiome shared across all statuses but was most abundant in early pregnancy (7.3%), while mid pregnancy also had a single indicator, *Unclassified Nitrincolaceae* (0.607). Late pregnancy had the most indicator species, with 31 identified, including *Unclassified Cellvibrionaceae* (0.753) and *Unclassified Enterobacterales_A_737866* (0.729) with the highest coefficients. There was also 1 species, *Haloferula harenae* (0.558), that was associated with the combination of early and late pregnancy.

### Similarities and differences between embryo and adult reproductive microbiomes

To investigate potential parental influences on embryo microbial community composition, we focused on samples from embryos, ovaries, pregnant, and non-pregnant brood pouches. A context for this investigation is the unique reproductive process by which females transfer eggs into the male’s brood pouch, providing potential opportunity for microbial transfer from male and female sources. Here, the fertilized eggs develop into embryos, remaining in the brood pouch until parturition. When comparing embryo and pregnant brood pouch sites, 58.7% of the taxa were shared (1,097 out of 1870 taxa). The embryos contained 220 unique taxa (11.8%), while pregnant brood pouches had 553 unique taxa (29.6%). In contrast, non-pregnant brood pouches and embryos shared only 40.3% of taxa (591 out of 1,468 taxa), with embryos having 726 unique taxa (49.5%) compared to 151 unique taxa (10.3%) in the non-pregnant brood pouches. This highlights that pregnant brood pouches and embryos may share more taxa, and that embryos may have more unique taxa compared to non-pregnant brood pouches. Ovaries and embryos shared 38% of their taxa (610 out of 1,606 taxa), with the ovaries hosting 289 unique taxa and the embryos containing 707 unique taxa. Ovaries and brood pouch samples shared 35.2% of taxa (688 out of 1,952 taxa), but ovaries contained 211 unique taxa (10.8%) and brood pouches demonstrated 1,053 unique taxa (53.9%).

**Fig. 5:**
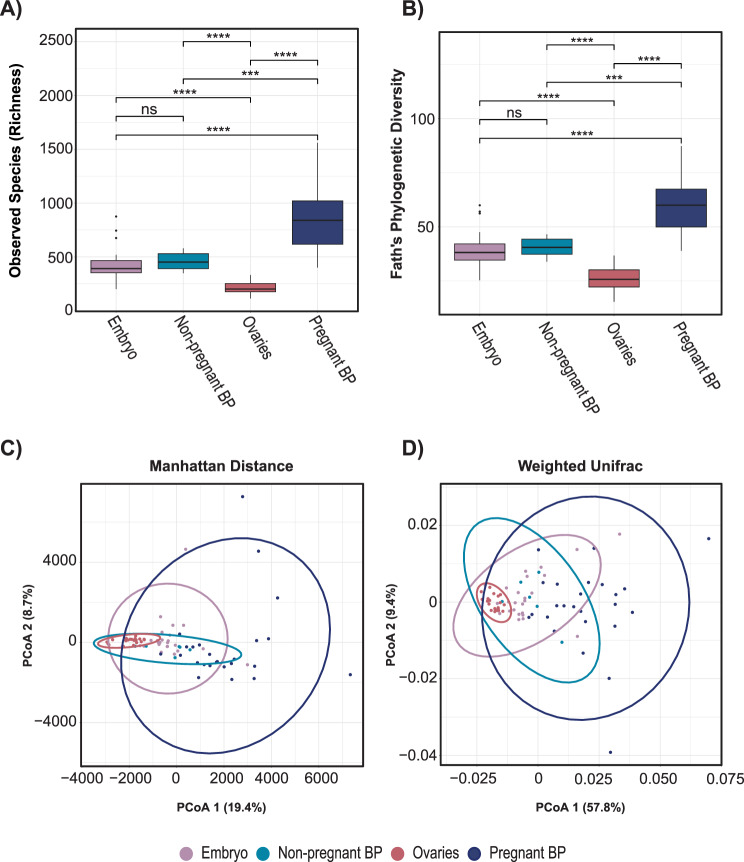
Alpha and beta diversity of microbiomes across the embryo, ovaries, pregnant brood pouch, and non-pregnant brood pouch, illustrating potential parental influence on the embryo’s microbiome. Alpha diversity, including (**A**) observed richness and (**B**) Faith’s phylogenetic diversity (PD), reveals differences in microbial richness and phylogenetic diversity across these sites. Beta diversity, shown with (**C**) Manhattan distance and (**D**) weighted UniFrac, highlights the dissimilarities in microbial community composition and phylogenetic relationships. Abbreviations: BP = brood pouch

In analyzing alpha diversity, significant differences were observed only in species richness and Faith’s PD (Fig. 5A–B). Embryos differed significantly from both ovaries and pregnant brood pouches but did not show significant differences when compared to non-pregnant brood pouches for both richness and Faith’s PD (Table S2). Ovaries had the lowest number of species and phylogenetic diversity, whereas pregnant brood pouches had the highest. The Shannon and inverse Simpson indices did not show significant differences between any of the parental or embryonal sites, indicating similar relative abundances and evenness of shared ASVs across these sites.

At the beta diversity level, bacterial community structure was distinct between all pairwise comparisons (pregnant brood pouch ×embryos, non-pregnant brood pouch ×embryos, pregnant brood pouch ×ovaries, non-pregnant brood pouch ×ovaries, ovaries ×embryos) when considering the Manhattan distance, although there is overlap in an ordination space of reduced dimensionality (Fig. 5C). All comparisons were significantly different based on PERMANOVA. In order of effect size (but not necessarily implying any ranking of biological significance), the pregnant brood pouch and ovaries exhibited the most pronounced difference at the beta diversity level (Table S3, PERMANOVA: R^2^ = 0.178, F = 9.324, *p* = 0.001), followed by non-pregnant brood pouch and ovaries (Table S3, PERMANOVA: R^2^ = 0.160, F = 4.557, *p* = 0.001) and ovaries and embryos (Table S3, PERMANOVA: R^2^ = 0.103, F = 4.934, *p* = 0.001). The non-pregnant brood pouch and embryo comparison showed a slightly lesser difference in beta diversity (Table S3, PERMANOVA: R^2^ = 0.083, F = 2.615, *p* = 0.001), followed by the pregnant brood pouch and embryo comparison (Table S3, PERMANOVA: R^2^ = 0.077, F = 4.001, *p* = 0.001). Analyses based on weighted UniFrac distance showed that the pregnant brood pouch and ovaries exhibited the most pronounced difference in beta diversity (Table S3, PERMANOVA: R^2^ = 0.454, F = 35.789, *p* = 0.001). This was followed by the pregnant brood pouch and embryos (R^2^ = 0.237, F = 14.874, *p* = 0.001), the non-pregnant brood pouch and ovaries (R^2^ = 0.218, F = 6.674, *p* = 0.002), and the ovaries and embryos (R^2^ = 0.213, F = 11.672, *p* = 0.001). In contrast, the comparison between the non-pregnant brood pouch and embryos was not significant (R^2^ = 0.058, F = 1.794, *p* = 0.085), suggesting greater similarity in their phylogenetic diversity and microbial abundance.

### Pregnant brood pouch and embryo source tracking analysis using fast expectation-maximization microbial source tracking (FEAST) suggests stronger paternal contributions to the male pregnancy microbiome

**Fig. 6:**
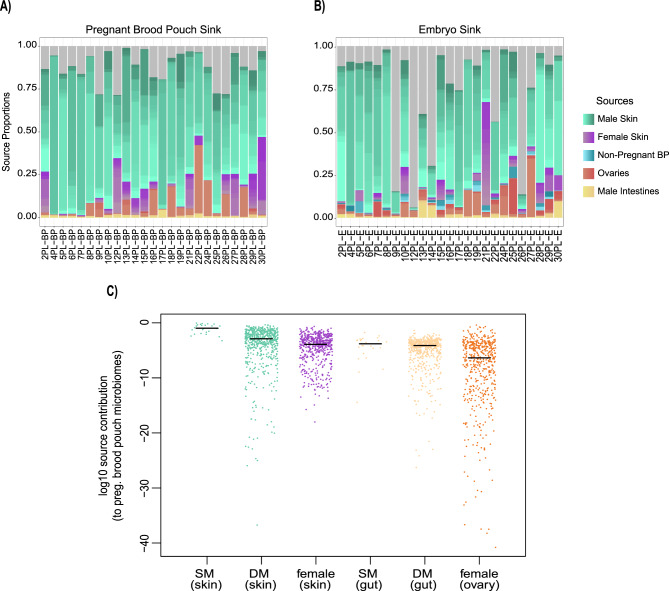
FEAST source tracking analysis. (**A**) pregnant brood pouch samples (*n* = 25) as the sink and (**B**) embryo samples (*n* = 25) as the sink. Sources include male skin (*n* = 31), female skin (*n* = 22), non-pregnant BP (*n* = 6) (only considered for embryo sink analysis), ovaries (*n* = 20), male intestines (*n* = 32), and unknown sources (grey). BP = brood pouch, E = embryos. (**C**) potential source sites for the male pregnancy microbiome differ in their average contribution, as estimated by FEAST. Shown are jittered log_10_ – transformed contribution proportions to 25 “sink” pregnant male brood pouch microbiomes. Each point represents the estimated contribution proportion from an individual “source” sample to one of the 25 “sink” samples. SM = same male (paternal); DM = different male (non-paternal). Black bars mark the medians of log_10_-transformed values for each group

We conducted microbial source tracking analysis using FEAST to investigate the potential microbial origins of the pregnant brood pouch microbiome and whether male body sites are more likely to contribute than female sites during male pregnancy. Pregnant brood pouch samples were considered sinks and male intestines, ovaries, male and female outer skin were sources (Fig. 6A). We categorized the analysis in this way because the brood pouch is in close proximity to the distal ovaries and maternal skin in that region during mating, and pregnancy is potentially influenced by the male’s skin near the brood pouch as well as the gut, which vents at the pouch anterior. Overall, the male outer skin source was found to be a primary source of bacteria in the pregnant brood pouch sites, with a 70.6% contribution ratio. This is followed by unknown sources (11.9%), female skin (9.3%), ovaries (6.8%), and male intestines (1.4%) as the smallest source proportions.

Source tracking with FEAST was also utilized to understand potential source contributions to the embryos from male and female sources (Fig. 6B). Embryo samples were considered the sink and male intestines, ovaries, male and female outer skin, and non-pregnant brood pouch samples were sources (Fig. 6B). Similar to the pregnant brood pouch source tracker analysis, the male outer skin source showed the largest source contribution (57.1%), followed by unknown sources (24.1%). Ovaries and female skin showed similar source contributions (7.8% and 6.4%, respectively), followed by non-pregnant brood pouch sources (2.5%), and lastly male intestines (2.1%).

We evaluated the distributions of individual source sample contributions to 25 pregnant male pouch microbiomes, including four body sites most likely to “seed” the male pregnancy microbiome: male skin, female skin, male intestine, and ovary. The distribution of FEAST contributions varied significantly across the four sources (Fig. 6; GLMM LRT = 270.21; *p* < 2.20e-16), with male skin samples demonstrating the highest contribution proportions, on average. Bootstrapped 95% confidence intervals for the median contribution proportions of each source type were as follows: male skin = 1.39e-3 (9.43e-4, 2.27e-3); female skin = 1.45e-4 (1.01e-4, 2.14e-4); male intestine = 8.28e-05 (6.94e-05, 1.08e-04); ovary = 4.32e-07 (1.27e-07, 1.35e-06).

To test for a clear signal of “same-male” (paternal) contributions in the case of male skin and male gut samples, we compared FEAST contribution values derived from the skin (intestine) of the same male to those derived from the skin (intestine) of different males. Interestingly, we found a significantly higher contribution score, on average, for paternal relative to non-paternal male skin samples (Fig. 6C; jackknife resampling medians test *p* < 0.001), but not for paternal relative to non-paternal intestine samples (Fig. 6C; jackknife resampling medians test *p* = 0.121), suggesting that male skin is a more likely route for vertical transmission of the microbiome via male pregnancy than the male gut.

### Bay pipefish microbiomes exhibit sexual dimorphism

**Fig. 7:**
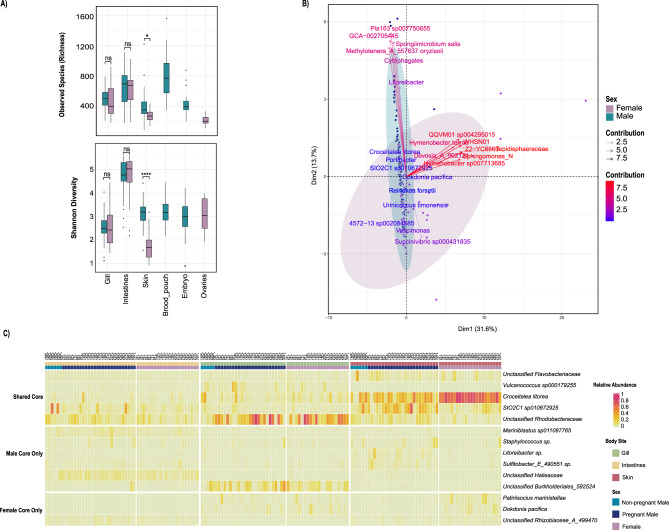
Comparison between the sexes considering all body sites (brood pouch, embryos, ovaries, gill, intestines, outer skin) with an emphasis on male and female differences in shared body sites (gill, intestines, outer skin). The alpha diversity metrics are (**A**) observed species richness and Shannon Index. (**B**) PCA biplot displaying indicator species contributions by sex (only considering shared sites). ASVs were only considered indicator species if *p* ≤ 0.001. (**C**) Heatmap of the core microbiome by sex and body site, organized by shared core species, followed by male-exclusive core, and then female-exclusive core. Statistically significant differences between groups are denoted using asterisk notation (*p* > 0.05 ns, *p* ≤ 0.05*, *p* ≤ 0.01**, *p* ≤ 0.001***, *p* ≤ 0.0001****)

To examine whether the bay pipefish microbiome is sexually dimorphic, we analyzed alpha and beta diversity by comparing only the body sites shared by males and females (gills, intestines, and outer skin), in addition to comparing all body sites for broader insights into overall diversity patterns. The former ensured that observed sex differences in diversity were not due to the inclusion of sex-specific tissues but instead reflect biological variation between male and female microbiomes arising from interactions with the same tissue and cell types. Alpha diversity between sexes when considering both comparisons (sex-shared body sites and all body sites) were all significantly different (Table S2, *p* ≤ 0.05) for observed richness, Shannon, Inverse Simpson, and Faith’s PD metrics. A more in depth look at the shared body sites showed that the outer skin was significantly different between males and females for all alpha diversity metrics including both observed richness and Shannon Index (Fig. 7A, Fig. S5 A-D). For all metrics, microbial diversity was significantly higher in male skin compared to female skin.

Based on the first two PcoA axes of both the Manhattan distance and weighted UniFrac analyses considering all body sites (Fig. S5 E-F), male and female samples on average occupied similar regions of space, but males and females were significantly different in microbial community composition considering the full multivariate space (Table S3, PERMANOVA: *p* = 0.001). For Manhattan distance, considering all body sites (Fig. S5E), sex accounted for about 3% of the dissimilarity (Table S3, PERMANOVA: R^2^ = 0.029, F = 7.073, *p* = 0.001), while the comparison of shared body sites alone explained about 2% (Table S3, PERMANOVA: R^2^ = 0.017, F = 2.772, *p* = 0.006); both were statistically significant. We included a body site-sex interaction term for the shared body sites analysis to test whether the effect of sex on the microbiome composition changed depending on the body site. While both main effects (sex and body site) were significant, the interaction was not significant (Table S3, PERMANOVA: R^2^ = 0.009, F = 0.908, *p* = 0.561) suggesting that the dissimilarity in microbial communities between males and females was consistent across all body sites. Similarly, for weighted UniFrac distance (Fig. S5F), the analysis including all body sites was significant and explained the same level of dissimilarity (Table S3, PERMANOVA: R^2^ = 0.029, F = 6.886, *p* = 0.001). However, when only shared body sites were considered, sex did not significantly affect the microbial community composition (Table S3, PERMANOVA: R^2^ = 0.007, F = 1.152, *p* = 0.260), indicating that overall microbial composition, when accounting for phylogenetic relationships and microbial abundance, does not differ by sex across shared sites. Body site remained a significant factor, indicating distinct microbial communities are driven by site-specific factors. PERMDISP suggested no significant differences by sex in dispersion for either the Manhattan or Weighted UniFrac distance of the shared body sites comparison.

### Core microbiome and indicator species analysis provide insights into sex-biased community members

A more in-depth core microbiome analysis of shared body sites between the sexes revealed 11 core ASVs for males and 8 for females (Fig. 7C). The sexes share 5 core ASVs including *Vulcanococcus sp000179255*, *Croceitalea litorea*, *Unclassified Flavobacteriaceae*, *SIO2C1 sp010672925*, and *Unclassified Rhodobacteraceae*. Overall, *Unclassified Flavobacteriaceae*, *SIO2C1 sp010672925*, and *Unclassified Rhodobacteraceae* are slightly more abundant in males (1.68% vs 1.26%, 8.95% vs 3.09%, and 13.08% vs 12.89% mean relative abundances, respectively) but are present in all male and female shared body site samples. *Vulcanococcus sp000179255* is more abundant in males (1.02% vs. 0.68%) but is slightly more prevalent in female samples (100% vs. 97.85%). In contrast, *Croceitalea litorea* is significantly more abundant in females (23.23% vs. 9.20%) and is present in all female samples compared to 98.92% of male samples. On a site-by-site basis regardless of sex, *Croceitalea litorea* and *SIO2C1 sp010672925* are most abundant in the outer skin samples, whereas *Vulcanococcus sp000179255* and *Unclassified Rhodobacteraceae* are more abundant in the gills, indicated by the darker boxes in the heatmap (Fig. 7C). There were 6 taxa unique to the male core microbiome and 3 taxa unique to the female core microbiome.

Indicator species analysis was used to elucidate ASVs that are strongly associated with males or females. Only sex-shared body sites (gills, intestines, and skin) were considered to focus on differences between the sexes that are not confounded by sex-specific sites. Males had 7 indicator species, while females had 16 (Fig. 7B, Fig. S6B). Among the male-associated indicator species, 2 ASVs, *SIO2C1 sp010672925* and *Litoreibacter sp.*, were also identified in the male core microbiome, suggesting these species may play an important role in the male microbiome. *SIO2C1 sp010672925* had the highest indicator value (0.284) for males, followed by *Spongiimicrobium salis* (0.243), *Unclassified GCA-002705445* (0.223), and *Methylotenera_A_557637 oryzisoli* (0.221). *SIO2C1 sp010672925* was classified as an indicator species for males, yet it was present in every shared body site sample from both males and females, though more abundant in males. Additionally, it was part of the core microbiome across all body sites. Female samples included 2 ASVs, *Croceitalea litorea* and *Dokdonia pacifica*, that had the highest indicator values for females (0.286 and 0.270, respectively) and were also shared between the core and indicator species analyses, due to being both highly prevalent and more abundant in females than males. *Reinekea forsetii* and *Vescimonas sp.* were both indicator species for females but were not present in males, which suggests that these species are biologically specific to female pipefish hosts.

## Discussion

### Male pregnancy microbiome diversity and potential function

Previous research demonstrated that the syngnathid brood pouch microbiome composition and diversity changes over the course of pregnancy [14]. Based on males sampled directly from a seagrass bed in their natural habitat, we found that the bay pipefish brood pouch microbiome diversity and composition significantly changes when comparing non-pregnant to pregnant developmental states. However, we found little evidence for microbiome changes throughout pregnancy. One possible explanation for the increased diversity at the onset of male pregnancy is that the *Syngnathus* brood pouch, characterized by its two pouch folds that seal along the midline without permanently fusing, creates a dynamic microenvironment [28, 29]. During breeding, the female deposits her eggs directly into the brood pouch, where they are fertilized by the male. This process likely introduces environmental microbes into the pouch, as well as maternal and paternal microbes, potentially facilitating microbial colonization during early pregnancy and maintaining this diversity into the later stages. The nutrient-rich microenvironment, resulting from biomolecules supplied by the male, may further support microbial growth [30].

Other mechanisms of male-mediated increase in pouch microbiome diversity during pregnancy could involve modulation of the host immune response, which is a well-documented phenomenon in the gut of animal hosts [31, 32], and is consistent with known transcriptomic changes in syngnathid brood pouch tissues during pregnancy [33–35]. The immune response in Syngnathidae has been well studied, showing that the brood pouch expresses many immune molecules more abundantly during pregnancy, with pregnant males exhibiting greater innate immune system activity than non-pregnant males [30, 34–38]. This enhanced immune activity may shape distinct microbial niches, promoting the establishment of beneficial symbionts that aid in protecting the developing embryos during male pregnancy and after birth when exposed to the environment as free swimming larvae. This idea is supported by studies showing that immunologically activated *Syngnathus typhle* parents, injected with heat-killed pathogens, exhibited significant shifts in microbiome composition and diversity, particularly in the late pregnancy brood pouch [14]. This immune activation also led to differentially expressed immune genes in the offspring and successfully reduced the relative abundance of experimentally introduced bacteria [39]. Together, these results suggest that parents can vertically transfer immunological experience to their offspring, enhancing their defense and survival against specific pathogens encountered after birth in the external environment.

Additionally, androgen-mediated responses to male pregnancy could help explain shifts in microbiome composition as they are known to modulate the host microbiome by altering mucosal immunity, barrier function, and microbial metabolism [40]. In male seahorses and pipefish, androgens decrease when males become pregnant, a change thought to optimize embryo survival [41]. Maternal hormones derived from the eggs, such as estrogens or progestins, may also enter the pouch and contribute to hormone-microbiome interactions that potentially support early immune modulation that influences the developing embryo’s microbial community. Similar hormonal shifts in other species have been shown to influence microbiome diversity and composition, providing a potential explanation for the microbiome alterations observed in the brood pouch during pregnancy [42].

Microbiome manipulations in pipefish have been performed experimentally, with various forms of evidence for differential embryo survivorship [13], but specific links between alpha diversity and brood fitness remain to be shown. Based on information about inferred bacterial taxa that likely contribute to the increase in pouch microbiome alpha diversity and differences in microbial community composition from a non-pregnant to pregnant state, we speculate on some potential functions relevant to host biology. For instance, we identified *Cognatiyoonia koreensis* and *Sulfitobacter_E_490551 sp.* as key species in both core and indicator analyses for pregnancy stages. *Cognatiyoonia koreensis* was found to be a core and indicator species in the non-pregnant brood pouch, suggesting its high fidelity to this stage. *Sulfitobacter_E_490551 sp.*, a core species across all pregnancy stages, was also identified an indicator species in the early pregnancy stage, indicating its potential role in driving the increased diversity observed during this period. This species, enriched in early pregnancy relative to non-pregnancy, is known for producing bioactive metabolites and algicidal properties [43]. The identification of these species in both core and indicator analyses underscore their potential importance in facilitating initial microbial colonization of the embryos from the non-pregnant to early pregnancy state, contributing to the observed shifts in diversity and composition.

Tanger et al. [12] reported similar brood pouch microbiome diversity results in *Syngnathus typhle*, specifically that alpha diversity was not affected by pregnancy stage among pregnant males. However, in contrast to our results, both Tanger et al. and Beemelmanns et al. [14] found that beta diversity was significantly different between the stages, mainly driven by a shift during late pregnancy. Although our analysis using frequency-based (Manhattan distance) and phylogenetic (weighted UniFrac distance) microbial community dissimilarity metrics suggested that early, mid, and late pregnancy brood pouch microbiomes were not significantly different from each other, we observed late pregnancy having the highest mean richness and phylogenetic diversity. Late pregnancy brood pouches also harbored the greatest number of indicator species compared to earlier pregnancy stages, a pattern consistent with findings from the same study [12]. It is hypothesized that before parturition, the brood pouch becomes more permeable, allowing its pouch fluid osmolality to align with the surrounding environment for embryo acclimation [44, 45]. This increased permeability may facilitate a greater exchange of microbes between the brood pouch and its external surroundings, possibly explaining the observed increase of both richness and phylogenetic microbial diversity and expansion of indicator species during late pregnancy in the pouch.

Previous research identified *Kiloniella sp.* as an indicator species during late pregnancy in *S. typhle* [14], and our study also found it to be a core microbe at this stage. As pregnancy progressed, *Kiloniella sp.* increased in both prevalence and abundance, with non-pregnant brood pouches showing the lowest abundance (0.02%) and prevalence (50%), while late pregnancy samples had the highest abundance (4%), and was present in every brood pouch and embryo sample. *Kiloniella* has been documented to reduce nitrate to nitrite [46] which could indicate an important role in managing nitrogenous waste produced by developing embryos. Similarly, *Ulvibacter* was found to be an indicator species during late pregnancy in the same study, although our study did not classify it as core or indicator. However, we found *Ulvibacter* had the highest prevalence in late pregnancy brood pouches (75%), despite maintaining a similar abundance to the non-pregnant state. The observation of these microbial species in multiple syngnathid studies suggests that both *Kiloniella* and *Ulvibacter* may play crucial roles in male pregnancy and embryo development, particularly during late pregnancy. This may reflect a selective process by males to sustain these beneficial bacteria at the end of pregnancy.

### Sources of the male pregnancy microbiome

We used source tracking analysis to explore the potential microbial origins of the embryo and pregnant brood pouch microbiomes, namely which parental sites provide the most likely contributions to microbial communities within the pouch during pregnancy. Our analysis suggests that the embryo’s microbial community is sourced from a variety of adult body and unknown origins, the most likely contribution being from male skin (Fig. 6B). This suggests that the male’s outer skin is a significant source of microbes for embryos, possibly transferred to embryos through vertical transmission, while maternal transmission through the ovaries and external sources such as environmental microbes contribute to embryonal microbiomes to a lesser extent. Another source tracking analysis study investigating the origins of the embryo microbiome in *Syngnathus typhle* reported that paternal microbial contributions mainly sourced the whole offspring microbiome and maternal contributions mainly sourced the offsprings’ internal microbiome over the course of pregnancy [12]. Although that study did not consider the outer skin as a potential microbial source, the observed trend of stronger male contributions to external embryo microbiomes in syngnathids aligns with our findings. In our case, we analyzed whole embryos without surface sterilization or dissection, which may reflect a mix of internal and external microbial sources. This likely bias towards paternal contributions to the external brooded offspring’s microbiome suggests that the father may be selecting for specific functionally important microbes that colonize the embryos, potentially to increase embryo fitness. One study supporting this idea found that offspring survival improved when paternal brood pouches were exposed to a cocktail of five bacterial strains associated with transgenerational transfer and early microbial colonization [13]. Notably, some of the most beneficial strains were isolated from paternal sources, highlighting the potential importance of paternally derived microbes in promoting offspring survival.

Similar to the embryo microbiome, the male outer skin was also the most likely source of bacteria for pregnant male brood pouch tissue, followed by unknown sources. Interestingly, paternal outer skin samples showed significantly higher contribution values than non-paternal male skin samples, suggesting that the main source of pregnant pouch-associated microbes is actually paternal in origin, and that microbes associated with the male’s outer skin near the brood pouch may play a significant role in shaping the brood pouch microbiome during pregnancy via vertical transmission. Similarity between the brood pouch and male outer skin microbiomes, and consequently at least some of the inferred signal from the source tracking analysis, may have arisen from the sampling methodology and represents a limitation of this analysis. During dissection, the entire brood pouch was collected, including its outer surface, which is exposed to the environment. As a result, microbes from the external skin and the exterior regions of the brood pouch samples may be expected to overlap due to this shared environmental exposure. While both the brood pouch and outer skin sites are exposed to the external environment, the brood pouch skinfolds that cover the eggs are histologically and morphologically distinct from the other epidermal regions of the fish, the morphology of the pouch in general changes drastically between brooding and non-brooding stages, and the inner brood pouch harbors placental tissues that function as egg compartments [11]. These distinct structural differences could create unique environmental niches for functionally specialized microbial communities, which is why the entire brood pouch was included in the sample. Although including the outer skinfolds of the brood pouch could be the reason for some observed similarity with skin samples, it is important to recognize that the outer skin and brood pouch microbiomes were nevertheless distinct with respect to alpha and beta diversity. To better understand the mechanisms of vertical transmission between the external skin and the pouch, future studies could include molecular tagging techniques such as fluorescence or isotopic labeling to trace the movement of specific bacteria between these tissues.

“Unknown sources,” which represent the proportion of a sink microbiome that cannot be attributed to defined source categories, were the second-most likely contribution class for both embryo and pregnant pouch sample types. These unmeasured contributions are most likely from environmental sources or contamination. One caveat of this study is the absence of environmental controls, such as surrounding water microbial communities. Without these samples, we acknowledge that this may introduce bias, particularly in fully distinguishing between microbes that are environmentally acquired and those that are truly host-associated. While our source tracking analyses allow us to explore likely parental and site-specific microbial contributions, future studies incorporating environmental microbial baselines would strengthen conclusions about microbial contributions to the male pregnancy microbiome.

### Microbiome diversity across body sites

The microbial community composition was significantly different across body sites (brood pouch, gills, intestines, outer skin, embryos, and ovaries) considering abundance-based and phylogenetic distances, and body site was the factor that explained most of the overall variation in community dissimilarity among samples. This conclusion is supported in other teleost species as well as humans, where microbial community composition was determined primarily by body site [47–49]. These compositional differences most likely indicate that the pipefish host may act as a selective ‘filter’, where each body site provides a distinct physiological and ecological environment that supports specific microbial communities. Such body site specific differences are likely a result of the unique environmental factors (e.g., pH, nutrient availability, temperature, adhesion proteins, mucus type) and other host selective mechanisms (e.g. immune system activity) present at each body site [50, 51].

Overall, the most abundant bacterial phyla in the bay pipefish microbiome, namely Proteobacteria, Bacteroidota, Actinobacteriota, and Firmicutes, align with findings from other teleost microbiome studies, which identified these phyla as predominant in the teleost skin and gut [4, 52]. This pattern is also consistent with other Syngnathidae research, where the dominant phyla in the Barbour’s seahorse (*Hippocampus barbouri*) and the broad-nosed pipefish (*Syngnathus typhle*) included Proteobacteria, Firmicutes, Bacteroidota, and Planctomycetota [14, 53]. Proteobacteria, highly prevalent in marine environments, contribute to fish growth through nutrient cycling and organic compound mineralization, while Bacteroidota are involved in fermentative metabolism and oligosaccharide degradation [54, 55]. The recurrent dominance of these phyla across various studies suggests a long evolutionary history of association with internal and external teleost host environments.

Core microbiome analysis to identify taxa with consistent representation within site types revealed 3 agglomerated ASVs that are core species in every body site: *Croceitalea litorea*, *SIO2C1 sp010672925*, and *Unclassified Rhodobacteraceae*. These taxa consistently appeared in multiple analyses, including as shared core species between males and females and across pregnancy stages in addition to being a core species for all body sites. Their repeated occurrence and high prevalence in the brood pouch tissues suggests they may play a fundamental role in the bay pipefish microbiome, especially during pregnancy. The specific functions of *Croceitalea litorea* are not well understood, but other species within the genus *Croceitalea* encode enzymes for metabolizing polysaccharides and proteins [56]. The strain *SIO2C1 sp010672925,* within the Cyanobacteria phylum, suggests it may play a role in nitrogen fixation and primary production within these body sites [57]. The *Rhodobacteraceae* family is highly ubiquitous in marine ecosystems, making up to 30% of bacterial communities [58]. Its presence in other syngnathid microbiome studies [14, 15] is not surprising, given its widespread occurrence, but this consistent finding may indicate its potential significance in the syngnathid microbiome. As *Croceitalea litorea* and *SIO2C1 sp010672925* are highly prevalent and abundant in the bay pipefish microbiome but not detected in other known syngnathid microbiome studies [14–17, 53, 59], these bacteria could be a product from the Oregon coastal environment or specific to the *Syngnathus leptorhynchus* microbiome.

Considering all the surveyed body sites, the brood pouch had the highest richness and phylogenetic diversity but had similar richness and evenness with the intestines. Previous studies have shown that the teleost gut microbiome is associated with many important functions supporting host health including nutrient absorption, immunity, and digestion [60]. While both the brood pouch and intestines exhibit the highest alpha diversity, one might consider that, similar to the functionally important gut microbiome, the brood pouch could host a specialized microbiome that supports and aids in a healthy pregnancy. Consistent with observations in *S. typhle*, where the placental-like brood pouch structure had the highest phylogenetic diversity (Faith’s PD) [12], our results on the bay pipefish brood pouch microbiome also showed high phylogenetic diversity, suggesting potential functional importance [61].

We found that the female ovary and male brood pouch microbiomes were compositionally distinct, a result that aligns with previous studies reporting distinct microbiomes in both the maternal gonads and paternal brood pouches of *S. typhle* [14]. Their study also identified *Marinomonas* as the most abundant bacterium in these reproductive sites of S*. typhle* in the Eastern Atlantic Ocean. However, in our study on *Syngnathus leptorhynchus*, which inhabits the Eastern Pacific Ocean, *Marinomonas* was not identified as a core or indicator species for any reproductive site and was absent in the ovaries but showed high prevalence in the embryos (80%) and brood pouch (71%), with mean relative abundances of 5 and 1%, respectively. This genus is known as an important initial colonizer of fish larvae [62, 63], aligning with our findings of its prevalence in embryo samples. The absence of *Marinomonas* in the ovaries suggests a paternal influence, likely crucial for larval development during pregnancy. Despite inhabiting different geographic regions, both *Syngnathus* species harbor this bacterium in their embryos, suggesting that the male host may select for this bacterium due to its importance for larval development. Another study reported that halibut larvae showed the highest survival when both *Pseudoalteromonas* and *Marinomonas* were present [63], and the presence of *Marinomonas* in our embryos and brood pouch, as well as in *S. typhle*, suggests it may play an important role in embryonic protection and development during male pregnancy.

The ovary microbiome had the lowest richness and phylogenetic diversity, as well as low beta diversity compared to the other body sites. The selective environment within the ovaries, coupled with their isolation from external environmental influences, might limit the range of microorganisms that can colonize, resulting in more similar communities across individuals and a lower number of microbial species within each sample. A study on a European pipefish species, *S. typhle*, found the ovaries to have the highest microbial diversity compared to the brood pouch across various stages [14]. This differing result may be attributed to the sampling method or extraction protocol used, as our study extracted DNA directly from tissue, whereas the other study employed a swabbing method, which tends to increase community richness compared to tissue biopsies [64, 65]. Surveying the ovary microbiome in other sygnathid species may help elucidate whether it is a site with high diversity or one prone to DNA extraction bias and contamination.

### Sexual dimorphism in the microbiome

Sexual dimorphism with respect to many different phenotypic traits is widespread in the animal kingdom and has been observed in *Syngnathus spp.,* where females are larger and more ornamented than males [66]. This dimorphism is also reflected in vertebrate immunity, where different immune responses between sexes likely contribute to sexual dimorphism of microbiomes [67–70]. Previous work has also shown that *S. typhle* exhibits sexual dimorphism in its microbiome [13]. Building on this, we wanted to investigate if the bay pipefish microbiome of males and females exhibits sexual dimorphism at shared body sites (gills, outer skin, and intestines). We found that the most pronounced difference in alpha diversity between male and female bay pipefish occurred in the outer skin body site, while the other shared body sites remain relatively similar. This sexual dimorphism in the outer skin is supported by the observed class differences in the relative abundance bar plot (Fig. 1C), where female skin is primarily composed of the class Bacteroidia, and male skin harbors a more class-rich and even microbiome. This clear sex difference in skin alpha diversity was also reflected at the ASV level, and it may indicate that the male host is selecting for specific bacterial communities on the outer skin via hormonal or immune mechanisms, perhaps providing a means for vertical transmission to offspring. One such bacterium, *Litoreibacter sp.*, identified as both a core and indicator species in males, shows the highest abundance and prevalence in male outer skin. Furthermore, *Litoreibacter sp.* was also a core species in the brood pouch and embryos, as well as a shared pregnancy stage core, making it a potential candidate for vertical transmission from paternal skin to offspring.

We also investigated the possibility that sex, and the interaction between sex and body site, might influence microbial community composition at the beta diversity level for shared body sites. Sexual dimorphism in overall community composition was subtle, restricted to analysis using frequency-based (Manhattan) distances, and we detected no significant sex-by-site interactions. At the level of overall community composition, we conclude that there is some evidence for sexual dimorphism that involves similar taxa across gills, outer skin, and intestines, but that these differences probably reflect sexual dimorphism for a small group of closely related microbes.

### Study limitations and future directions

Our study was subject to some limitations due to the focus on sampling wild-caught pipefish from a natural system, particularly regarding the sample size of some groups. Our sampling effort was restricted to one time point in the middle of the breeding season, which produced only four late pregnancy and six non-pregnant male representatives. The ability to collect more samples could have increased the power to detect significant differences during late pregnancy. Additionally, while one might expect the non-pregnant brood pouch to be a likely contributor to the embryo microbiome, it was among the least significant sources in our source tracking analysis, possibly due to the limited sample of non-pregnant brood pouches not fully capturing the microbial diversity of this site. As stated, our source tracking analysis would also have benefited from environmental samples to help delineate the contribution of unknown sources. The lack of environmental samples is a limitation of this study that could cause misinterpretation of source contributions. Future studies should include various environmental samples such as water, vegetation, and substrate to help quantify and decipher these contributions.

Furthermore, microbial variation across the exact pregnancy stages within each coarsely defined pregnancy group potentially influenced the observed increase in beta diversity between pregnant and non-pregnant groups. Since individual males could only be staged by the embryos’ general developmental progress rather than the exact number of days pregnant, their microbiomes may exhibit variation in community composition. For example, two samples classified as early pregnancy could differ due to slight differences in embryo development, introducing variability even within the early pregnancy stage.

Additionally, some similarity between the brood pouch and outer skin microbiomes, as well as between the brood pouch and embryos, may reflect limitations in sampling design that could introduce bias. The entire brood pouch was collected during dissection, including its outer surface, which is exposed to the environment. We acknowledge that sampling the entire brood pouch, including external skinfolds, may have introduced overlap with the male outer skin microbiome. However, as detailed in the main text, both structural differences and observed differences in microbial diversity and community composition support that the brood pouch is distinct from the outer skin site. We also used whole embryos for microbial analysis, including both surface-associated and internal microbes. Since the embryos were collected directly from the brood pouch, similarity between their microbiomes may reflect both biological connection and shared sampling. While this approach may limit our ability to separate parental contributions, significant differences in alpha and beta diversity between pregnant brood pouches and embryos indicate that they harbor distinct microbial communities despite being sampled from the same environment.

Microbial profiling using 16S rRNA gene sequencing has known limitations, including sequencing platform and extraction protocol bias, unreliability of taxonomic classification at the species level, and variability in results due to the use of different analytical pipelines [71]. As sequencing technology advances, future syngnathid microbiome studies should include long-read sequencing to aid in species level annotation and incorporate a multi-omic approach to help elucidate functional information about the microbes present, especially during pregnancy.

## Conclusions

Our study provides a comprehensive analysis of the bay pipefish (*Syngnathus leptorhynchus*) microbiome and is the first study to characterize the wild microbiome of multiple body sites in a syngnathid species from its natural environment. We identified distinct microbial communities related to sex-specific body sites critical to the reproductive biology of the host, specifically unique microbiome variation associated with the phenomenon of male pregnancy. These insights include the microbial community composition of the male brood pouch, which is distinct and highly diverse, potentially aiding in embryonic protection and development during pregnancy. Additionally, the source tracking analysis suggested a predominant paternal influence on the embryo and pregnant brood pouch microbiomes, setting the stage for male pregnancy as a mechanism of vertical transmission of beneficial microbes. The study also provides evidence for sexual dimorphism in the microbiome, particularly among outer skin samples. Overall, these insights into the bay pipefish microbiome not only enhance our understanding of microbial diversity in syngnathids but also open avenues for future research into the microbiome’s functional roles in the evolutionary novelty of male pregnancy.

## Materials and methods

### Sample collection

A total of 54 bay pipefish were collected on July 17 and July 18, 2021, from Coos Bay, Oregon, consisting of 25 pregnant males (13 early pregnancy, 8 mid pregnancy, and 4 late pregnancy), 6 non-pregnant males, and 22 females. Male individuals had an average weight of 3.95 [g] while female individuals had an average weight of 2.51 [g]. The pipefish were caught with a seine net and were sorted into male and female buckets. Each individual was placed in a Falcon tube and flash frozen immediately using dry ice. Samples were then stored at −80 °C until being shipped (on dry ice) to the University of Idaho for dissection and DNA extraction. The males’ brood pouch (whole brood pouch which includes internal and external tissue), gills, gastrointestinal tract, outer skin posterior to the brood pouch, and embryos (in pregnant males only, whole embryos were used including both surface associated and internal microbes), and the females’ ovaries, outer skin posterior to the ovaries, gills, and gastrointestinal tract were dissected using aseptic techniques, totaling 237 body site samples. There were no signs of disease or infection observed during the dissection of these samples. Embryo samples were visually classified by developmental stage (13 early, 8 mid, and 4 late pregnancy) using the methods and descriptions outlined by Whittington et al. and Sommer et al. [33, 72]. Specifically, early embryogenesis was characterized by the presence of early cleavages, embryonic shield, and primitive streak features. Mid development included eye formation and pigmentation, while late development was defined by the formation of the snout and ventral jaw. These tissue samples were then used for DNA extractions as described below.

### DNA extraction, library prep, and sequencing

First, tissues were homogenized following a 2-step homogenization protocol. Briefly, tissue samples were transferred to 2 mL tough microtubes (2.4 mm metal beads, certified free of microbial DNA, OMNI International, Kennesaw, GA, USA) with 800 ul ATL (Qiagen, Valencia, CA, USA). Samples were then homogenized at 6 m/s for two 45-second cycles in the OMNI Bead Ruptor Elite Bead Mill Homogenizer, using the OMNI Bead Ruptor Cryo Cooling Unit to prevent heat-induced DNA degradation. For the second homogenization step, the entire homogenate was transferred into 2 mL microtubes (0.5 mm glass beads, certified free of microbial DNA, OMNI International, Kennesaw, GA, USA) and homogenized again under identical parameters as the initial step. Microbial DNA was then extracted following the Qiagen DNeasy spin column protocol described by Small et al., 2019 [73]. DNA was submitted to the University of Oregon Genomics Core Facility (GC3F) for 16S rRNA amplicon library construction and sequencing with Illumina NovaSeq 6000 sequencing (paired-end 150 bp reads). The hypervariable V4 region of the 16S rRNA gene was PCR amplified using the primers 515F and 806 R [74] during library construction. Several negative controls (no DNA template) were included during PCR amplification and library preparation to identify potential contaminants. Additionally, three ZymoBIOMICS Microbial Community Standards (Zymo Research, USA) were used as positive controls during sequencing.

### Statistical analysis

The raw FASTQ files were imported into QIIME2 [75] (version 2024.2) and demultiplexed using ‘*demux*’ (emp-paired). Sequences went through quality control with ‘*qiime quality-filter q-score*’ using the default settings where low quality sequences were filtered out. Paired reads were merged, denoised, and clustered into amplicon sequence variants (ASVs) with Deblur [76] (*–p-trim-length 145*), which also removes singletons, PhiX sequences, adapter sequences, and chimeric sequences during the denoising process. ASVs were classified using the naïve Bayesian classifier pre-trained on the Greengenes2 (version 2022.10) from 515F/806 R (V4) region of sequences [77, 78]. A phylogenetic tree was generated with the FastTree pipeline [79] (‘*align-to-tree-mafft-fasttree*’) using the representative sequences from the Deblur output. The ASV table was filtered to exclude mitochondrial, chloroplast, and unassigned reads, as well as taxa that only had a domain level annotation. All analyses after this point were performed using R [80] (version 4.3.2). The QIIME2 artifacts were converted to phyloseq objects with qiime2R (version 0.99.6) and we identified contaminants with decontam [81] (version 1.22.0). Those ASVs considered contaminants (Supplemental Table S1) were removed from the table for downstream analysis. ASVs represented by only one read in the entire dataset, as well as taxa present in only a single sample, were removed using the Phyloseq [82] (version 1.46.0) and genefilter [83] (version 1.84.0) packages. Thus, taxa were retained if they had more than one read in total and were present in at least two samples. All samples with less than 40,000 reads were removed from the table, resulting in the exclusion of two ovary samples and the negative controls.

### Alpha and beta diversity

A proportional normalization method, using the median library size as a multiplier, was applied to the ASV table for alpha diversity analysis. Alpha diversity was calculated using Observed Richness, Shannon, and Inverse Simpson indices as implemented in the vegan R package [84] (version 2.6.4) and Faith’s Phylogenetic Diversity (Faith’s PD) in the biomeUtils R package [85] (version 0.021). Overall group comparisons were conducted using Kruskal–Wallis tests, while pairwise significance tests between groups of interest were performed using the Wilcoxon test with Holm-corrected p-values to account for multiple comparisons. Beta diversity was assessed using community-level comparisons, with ASV raw abundance values normalized to logCPM (*cpm function, log = TRUE, prior.count = 0.5*) using the edgeR package [86] (version 4.0.16) to account for differences in sequencing depth. Principal coordinates analysis (PCoA) based on Manhattan Distance [87] and Weighted UniFrac [88] was used to visualize microbial community dissimilarity, and differences in overall community composition were assessed using permutational multivariate analysis of variance (PERMANOVA) with the ‘*adonis2*’ function in vegan, and pairwise PERMANOVA with the ‘*pairwise.adonis’* function with Holm’s adjusted p-values in the pairwiseAdonis R package [89] (version 0.4.1), applying 999 permutations. To determine if differences in beta diversity were influenced by differences in dispersion within groups, we employed permutational analysis of multivariate dispersions (PERMDISP) using vegan’s ‘*betadisper*’ and ‘*permutest*’ function with 999 permutations.

### Indicator species, core, and source tracking microbiome analysis

Indicator species analysis, using the indicspecies package [90] (version 1.7.14), identified amplicon sequencing variant (ASVs) strongly linked to specific tissue types by assessing their relative abundance and site fidelity. We utilized the proportional normalization ASV table and applied the ‘*multipatt*’ function with 999 permutations. For body site and sex comparisons, we considered only ASVs with an alpha level of 0.001, while for pregnancy stage and status comparisons, an alpha of 0.01 was used to allow for the identification of more species. The point-biserial correlation coefficient (*r.g*) was used to quantify the strength of association between species and groups of interest. To provide a more accurate representation of the core microbiome, the ASV table was merged using ‘*tax_glom’* function by species with ‘*na.rm = FALSE’* from the phyloseq package which resulted in 2,605 taxa. This agglomeration process consolidates taxa with identical taxonomic classifications, thus providing a more accurate reflection of their abundance and prevalence based on the current taxonomic database information available. However, it is important to note that limitations in the databases still exist, potentially affecting the precision of these classifications, especially at higher taxonomic classifications like class and order. The core microbiome was identified using the ‘*core*’ and ‘*core_members*’ functions from the microbiome R package (version 1.24.0) [91], with a prevalence (i.e., the number of samples in which they are present) threshold of 50% and a detection threshold of 0.001. Only agglomerated ASVs among the top 25 most abundant in the group of interest (e.g., body site, sex, pregnancy stage, or status) were considered. The chosen core microbiome parameters were consistent with other microbiome studies [92–94]. As environmental samples were not included, we recognize that some core taxa may originate from external sources. Differentially abundant core and indicator taxa were calculated using Analysis of Compositions of Microbiomes with Bias Correction [95] (ANCOM-BC) in QIIME2 using the parameter *‘–p-significance-threshold 0.01*’ that has been corrected using the Holm’s method (Supplemental Results) [96].

To quantify the proportional contributions of different microbial samples (sources) to both embryos and pregnant brood pouch body site samples (sinks), we used fast expectation-maximization microbial source tracking (FEAST) implemented in the FEAST R package [97] (version 0.1.0). We used the default settings with ‘*different_sources_flag = 0’* to specify that sink samples were tested with the same group of sources. We used the FEAST contribution estimates from the pregnant brood pouch sink analysis to test whether 1 or more of 4 probable source categories (male skin, female skin, male gut, female ovary) are more likely contributors to the male pregnancy microbiome. We performed this test by fitting a generalized linear mixed model with sink (pregnant pouch sample) as a random effect and ‘*family = beta_family*,’ using the *glmmTMB* function from the R package glmmTMB [98] (version 1.1.10). Indicator species PCA biplots were plotted with factoextra (version 1.0.7) and FactoMineR (version 2.12) [99]. We also tested for stronger paternal relative to non-paternal contributions from male skin and male gut source samples, by comparing the median contribution from paternal (i.e. skin and gut from the same male) samples (*N* = 25) to a jackknifed distribution of medians (1000 iterations) from the non-paternal (skin and gut samples from different males) contribution scores (*N*_*skin*_ = 600, *N*_*gu*t_ = 600).

## Electronic supplementary material

Below is the link to the electronic supplementary material.

Supplementary Material 1

## Data Availability

Raw 16S amplicon sequencing reads were submitted to the Sequence Read Archive (SRA) under BioProject PRJNA1243998. Data (ASV sequences and sample metadata), software information, and code necessary to run the analyses conducted in this report are available via the following Figshare links: ASV sequences (https://figshare.com/s/e1d80195049307a3529b) Sample metadata (https://figshare.com/s/e997873555deeea04d7b) Analysis artifacts (https://figshare.com/s/1a04643dd9d0bcd2b81c) Indicator species analysis results spreadsheet (https://figshare.com/s/37edb68a3c039170265a)

## Abbreviations

ASV: Amplicon sequencing variant
PERMANOVA: Permutational multivariate analysis of variance
PERMDISP: Permutational multivariate analysis of dispersion
PCoA: Principal coordinates analysis
FEAST: Fast expectation-maximization microbial source tracking
PCA: Principal Component Analysis
