# Supplementary material for: Signatures and likely sources of the male pregnancy microbiome in wild bay pipefish (Syngnathus leptorhynchus)
Source: Anim Microbiome. 2025 Oct 27;7:112. doi: 10.1186/s42523-025-00476-y (PMC12560297; doi:10.1186/s42523-025-00476-y)
Supplement: Supplementary file 1 — Supplementary Material 1 [file 42523_2025_476_MOESM1_ESM.docx]

**Supplementary**

| **ASV** | **Kingdom** | **Phylum** | **Class** | **Order** | **Family** | **Genus** | **Species** |
| --- | --- | --- | --- | --- | --- | --- | --- |
| e446ed23e1d2738ab7e1d9d62c944e18 | Bacteria | Bacteroidota | Bacteroidia | Bacteroidales | Bacteroidaceae | Alloprevotella | sp900095835 |
| f599a218c929475781e92af2ce316994 | Bacteria | Bacteroidota | Bacteroidia | Bacteroidales | Paludibacteraceae | Paludibacter | propionicigenes |
| c990d03aa922daa97e6ad3bc12c19a83 | Bacteria | Bacteroidota | Bacteroidia | Chitinophagales | Saprospiraceae | Lewinella_A | aurantiaca |
| aa495f040470da511d2291e75f4dc69d | Bacteria | Actinobacteriota | Actinomycetia | Mycobacteriales | Pseudonocardiaceae | Saccharopolyspora_C_379454 | gloriosae |
| e9950a6d7bf28f6212ac54c900b45e49 | Bacteria | Actinobacteriota | Actinomycetia | Actinomycetales | Microbacteriaceae | Unclassified | Unclassified |
| 533feaa0d2d04e87325b9b207e7a4b48 | Bacteria | Proteobacteria | Alphaproteobacteria | Sphingomonadales | Sphingomonadaceae | Sphingomonas_L_486704 | Unclassified |
| fe988b498243f5763665496282e0640c | Bacteria | Proteobacteria | Alphaproteobacteria | Rhizobiales_A_504705 | Beijerinckiaceae | Methylobacterium | aerolatum |
| 78e75db23aa8826ad56702ed91f203fc | Bacteria | Proteobacteria | Alphaproteobacteria | Unclassified | Unclassified | Unclassified | Unclassified |
| 9706ace3d855e92304ebbcd3c7689e5b | Bacteria | Proteobacteria | Alphaproteobacteria | Rhizobiales_A_500471 | Rhizobiaceae_A_500471 | Agrobacterium | Unclassified |
| af785624048cbf268e8cc55d50147333 | Bacteria | Proteobacteria | Alphaproteobacteria | Micavibrionales | Micavibrionaceae | Unclassified | Unclassified |
| f0b2a8478e09a2e8e47d19bd72fa8b48 | Bacteria | Calditrichota | Calditrichia | Calditrichales | Calditrichaceae | Unclassified | Unclassified |
| 9423014b19cfb5758cde3ce6d636b03b | Bacteria | Proteobacteria | Alphaproteobacteria | Rhodobacterales | Rhodobacteraceae | Unclassified | Unclassified |
| 49f18e082d8e1870064ffae132c925de | Bacteria | Proteobacteria | Alphaproteobacteria | Rhodobacterales | Rhodobacteraceae | Roseobacter | Unclassified |
| 4e15c882a9d6432119d869ad8738d571 | Bacteria | Proteobacteria | Gammaproteobacteria | DSM-26407 | DSM-26407 | Thioalbus | Unclassified |
| 32a1bffdb4bc98c2b6c6484f54913ac9 | Bacteria | Proteobacteria | Gammaproteobacteria | Burkholderiales_592524 | Burkholderiaceae_A_580492 | Ralstonia | Unclassified |
| 7fc5198a727563f183e272eea46cd055 | Bacteria | Proteobacteria | Gammaproteobacteria | Burkholderiales_592522 | Unclassified | Unclassified | Unclassified |
| d8f146bea70c9552a86ee8331566d999 | Bacteria | Proteobacteria | Gammaproteobacteria | Unclassified | Unclassified | Unclassified | Unclassified |
| ab6963c91c7195e2421920e51a3451bd | Bacteria | Proteobacteria | Gammaproteobacteria | Unclassified | Unclassified | Unclassified | Unclassified |
| 49d2a1fafa303fab93c6aee576456e65 | Bacteria | Proteobacteria | Gammaproteobacteria | Unclassified | Unclassified | Unclassified | Unclassified |
| **Supplemental Table 1**. 19 microbial contaminants identified through the decontam R package using the frequency method. These ASVs were removed from the ASV table for downstream analysis.   \| **Groups** \| \| **Observed Richness** \| \| \| **Shannon** \| \| \| **Inverse Simpson** \| \| \| **Faith’s PD** \| \| \| \| --- \| --- \| --- \| --- \| --- \| --- \| --- \| --- \| --- \| --- \| --- \| --- \| --- \| --- \| \| *Effect Size* \| *Test Statistic* \| *p-value* \| *Effect Size* \| *Test Statistic* \| *p-value* \| *Effect Size* \| *Test Statistic* \| *p-value* \| *Effect Size* \| *Test Statistic* \| *p-value* \| \| **All Body Sites** \| All Body Sites \| 0.439 \| 105 \| **<0.001** \| 0.446 \| 107 \| **<0.001** \| 0.351 \| 85 \| **<0.001** \| 0.374 \| 91 \| **<0.001** \| \| BP x E \| 0.637 \| 676 \| **<0.001** \| 0.162 \| 461 \| 0.924 \| 0.076 \| 422 \| 1.0 \| 0.620 \| 669 \| **<0.001** \| \| BP x G \| 0.501 \| 1291 \| **<0.001** \| 0.428 \| 1220 \| **<0.001** \| 0.340 \| 1135 \| **0.020** \| 0.436 \| 1228 \| **<0.001** \| \| BP x I \| 0.182 \| 1020 \| 0.190 \| 0.678 \| 152 \| **<0.001** \| 0.597 \| 234 \| **<0.001** \| 0.306 \| 1146 \| **0.019** \| \| BP x O \| 0.837 \| 620 \| **<0.001** \| 0.051 \| 329 \| 1.0 \| 0.097 \| 346 \| 1.0 \| 0.835 \| 619 \| **<0.001** \| \| BP x S \| 0.715 \| 1528 \| **<0.001** \| 0.328 \| 1146 \| **0.024** \| 0.299 \| 1117 \| 0.056 \| 0.680 \| 1494 \| **<0.001** \| \| O x E \| 0.771 \| 476 \| **<0.001** \| 0.085 \| 225 \| 1.0 \| 0.007 \| 252 \| 1.0 \| 0.725 \| 463 \| **<0.001** \| \| O x G \| 0.685 \| 982 \| **<0.001** \| 0.336 \| 293 \| **0.035** \| 0.197 \| 387 \| 0.570 \| 0.683 \| 981 \| **<0.001** \| \| O x I \| 0.709 \| 1041 \| **<0.001** \| 0.637 \| 990 \| **<0.001** \| 0.548 \| 927 \| **<0.001** \| 0.668 \| 1012 \| **<0.001** \| \| O x S \| 0.452 \| 218 \| **<0.001** \| 0.242 \| 697 \| 0.240 \| 0.240 \| 696 \| 0.330 \| 0.423 \| 238 \| **0.002** \| \| G x I \| 0.356 \| 824 \| **0.001** \| 0.778 \| 136 \| **<0.001** \| 0.699 \| 265 \| **<0.001** \| 0.155 \| 1152 \| 0.112 \| \| G x S \| 0.418 \| 2046 \| **<0.001** \| 0.045 \| 1306 \| 1.0 \| 0.042 \| 1445 \| 1.0 \| 0.425 \| 2057 \| **<0.001** \| \| G x E \| 0.172 \| 511 \| 0.190 \| 0.260 \| 860 \| 0.160 \| 0.222 \| 829 \| 0.370 \| 0.222 \| 471 \| 0.104 \| \| I x S \| 0.592 \| 2414 \| **<0.001** \| 0.769 \| 2707 \| **<0.001** \| 0.708 \| 2607 \| **<0.001** \| 0.503 \| 2266 \| **<0.001** \| \| I x E \| 0.425 \| 316 \| **<0.001** \| 0.676 \| 105 \| **<0.001** \| 0.605 \| 165 \| **<0.001** \| 0.332 \| 395 \| **0.016** \| \| S x E \| 0.313 \| 921 \| **0.017** \| 0.165 \| 799 \| 0.730 \| 0.190 \| 819 \| 0.570 \| 0.245 \| 865 \| 0.092 \| \| **Sex** \| M x F \| 0.335 \| 3827 \| **<0.001** \| 0.154 \| 5225 \| **0.019** \| 0.170 \| 5100 \| **0.009** \| 0.335 \| 3829 \| **<0.001** \| \| **Sex Shared Sites** \| M x F \| 0.164 \| 2477 \| **0.039** \| 0.210 \| 2313 \| **0.008** \| 0.217 \| 2285 \| **0.006** \| 0.169 \| 2459 \| **0.033** \| \| M G x F G \| 0.186 \| 402 \| 0.364 \| 0.008 \| 333 \| 1.0 \| 0.003 \| 331 \| 1.0 \| 0.157 \| 391 \| 0.530 \| \| M I x F I \| 0.059 \| 376 \| 0.673 \| 0.022 \| 343 \| 1.0 \| 0.060 \| 327 \| 1.0 \| 0.139 \| 410 \| 0.530 \| \| M S x F S \| 0.366 \| 488 \| **0.024** \| 0.749 \| 643 \| **<0.001** \| 0.751 \| 644 \| **<0.001** \| 0.342 \| 479 \| **0.037** \| \| **Pregnancy Status** \| P x NP \| 0.376 \| 574 \| **<0.001** \| 0.007 \| 1433 \| 0.935 \| 0.016 \| 1412 \| 0.848 \| 0.412 \| 491 \| **<0.001** \| \| **Pregnancy Stage (Male Samples ONLY)** \| All Preg Stages \| 0.137 \| 24 \| **<0.001** \| 0 \| 1.708 \| 0.635 \| 0 \| 2 \| 0.678 \| 0.171 \| 28 \| **<0.001** \| \| NP x EP \| 0.489 \| 1251 \| **<0.001** \| 0.056 \| 815 \| 1.0 \| 0.062 \| 821 \| 1.0 \| 0.529 \| 1292 \| **<0.001** \| \| NP x MP \| 0.420 \| 694 \| **0.004** \| 0.059 \| 427 \| 1.0 \| 0.041 \| 437 \| 1.0 \| 0.478 \| 726 \| **<0.001** \| \| NP x LP \| 0.553 \| 379 \| **0.001** \| 0.026 \| 223 \| 1.0 \| 0.007 \| 228 \| 1.0 \| 0.590 \| 389 \| **<0.001** \| \| EP x MP \| 0.178 \| 1601 \| 0.202 \| 0.113 \| 1498 \| 1.0 \| 0.112 \| 1497 \| 1.0 \| 0.163 \| 1578 \| 0.280 \| \| EP x LP \| 0.081 \| 734 \| 0.760 \| 0.098 \| 749 \| 1.0 \| 0.078 \| 731 \| 1.0 \| 0.061 \| 715 \| 1.0 \| \| MP x LP \| 0.114 \| 456 \| 0.760 \| 0.018 \| 409 \| 1.0 \| 0.024 \| 412 \| 1.0 \| 0.085 \| 442 \| 1.0 \| \| **Brood Pouch Sites ONLY** \| P BP x NP BP \| 0.611 \| 7 \| **<0.001** \| 0.072 \| 67 \| 0.715 \| 0.135 \| 60 \| 0.478 \| 0.611 \| 7 \| **<0.001** \| \| BP x M S \| 0.676 \| 858 \| **<0.001** \| 0.028 \| 496 \| 0.834 \| 0.035 \| 461 \| 0.791 \| 0.620 \| 827 \| **<0.001** \| \| P BP x E \| 0.737 \| 581 \| **<0.001** \| 0.177 \| 377 \| 1.0 \| 0.103 \| 350 \| 1.0 \| 0.734 \| 45 \| **<0.001** \| \| NP BP x E \| 0.184 \| 55 \| 0.317 \| 0.081 \| 66 \| 1.0 \| 0.027 \| 78 \| 1.0 \| 0.126 \| 61 \| 0.510 \| \| P BP x O \| 0.851 \| 0 \| **<0.001** \| 0.055 \| 234 \| 1.0 \| 0.112 \| 217 \| 1.0 \| 0.851 \| 0 \| **<0.001** \| \| NP BP x O \| 0.716 \| 120 \| **<0.001** \| 0.036 \| 63 \| 1.0 \| 0.036 \| 63 \| 1.0 \| 0.704 \| 119 \| **<0.001** \| \| NP x EP \| 0.744 \| 76 \| **0.002** \| 0.221 \| 50 \| 1.0 \| 0.241 \| 51 \| 1.0 \| 0.724 \| 75 \| **0.003** \| \| NP x MP \| 0.656 \| 43 \| **0.050** \| 0.069 \| 22 \| 1.0 \| 0.138 \| 28 \| 1.0 \| 0.690 \| 44 \| **0.040** \| \| NP x LP \| 0.809 \| 24 \| **0.048** \| 0.067 \| 11 \| 1.0 \| 0.067 \| 11 \| 1.0 \| 0.809 \| 24 \| **0.040** \| \| EP x MP \| 0.269 \| 69 \| 0.714 \| 0.237 \| 67 \| 1.0 \| 0.190 \| 64 \| 1.0 \| 0.253 \| 68 \| 0.642 \| \| EP x LP \| 0.028 \| 25 \| 0.956 \| 0.275 \| 36 \| 1.0 \| 0.357 \| 39 \| 0.978 \| 0.110 \| 22 \| 0.703 \| \| MP x LP \| 0.343 \| 23 \| 0.714 \| 0.098 \| 14 \| 1.0 \| 0.196 \| 12 \| 1.0 \| 0.392 \| 24 \| 0.642 \| \| **Supplemental Table 2.** Alpha diversity metrics (Richness, Shannon, Inverse Simpson, and Faith’s Phylogenetic Diversity) with statistical comparisons made using the Kruskal-Wallis test for multiple groups and Mann-Whitney U test for pairwise comparisons. Holm’s correction was applied to adjust for multiple comparisons. Significant p-values (*P ≤ 0.05*) are bolded. Brood pouch = BP, Embryo = E, Gill = G, Intestines = I, Ovaries = O, Skin = S, Male = M, Female = F, Pregnant = P, Non-pregnant = NP, Early pregnancy = EP, Mid pregnancy = MP, Late pregnancy = LP. \| \| \| \| \| \| \| \| \| \| \| \| \| \|  \| **Groups** \| \| \| **PERMANOVA** \| \| \| \| \| \| **PERMDISP** \| \| \| \| \| \| \| --- \| --- \| --- \| --- \| --- \| --- \| --- \| --- \| --- \| --- \| --- \| --- \| --- \| --- \| --- \| \| **Manhattan** \| \| \| **Weighted UniFrac** \| \| \| **Manhattan** \| \| \| **Weighted UniFrac** \| \| \| \| $R^{2}$ \| *Pseudo-F* \| *p-value* \| $R^{2}$ \| *Pseudo-F* \| *p-value* \| \| *F* \| *p-value* \| \| *F* \| *p-value* \| \| **All Body Sites** \| All Body Sites \| \| 0.245 \| 14.824 \| **0.001** \| 0.474 \| 41.214 \| **0.001** \| \| 48.804 \| **0.001** \| \| 32.542 \| **0.001** \| \| BP x E \| \| 0.059 \| 3.387 \| **0.001** \| 0.172 \| 11.222 \| **0.001** \| \| 29.232 \| **0.001** \| \| 12.020 \| **0.001** \| \| BP x G \| \| 0.179 \| 17.714 \| **0.001** \| 0.307 \| 35.864 \| **0.001** \| \| 39.857 \| **0.001** \| \| 23.673 \| **0.001** \| \| BP x I \| \| 0.205 \| 21.454 \| **0.001** \| 0.350 \| 44.594 \| **0.001** \| \| 3.649 \| 0.062 \| \| 9.883 \| **0.001** \| \| BP x O \| \| 0.143 \| 8.147 \| **0.001** \| 0.368 \| 28.505 \| **0.001** \| \| 67.400 \| **0.001** \| \| 33.716 \| **0.001** \| \| BP x S \| \| 0.095 \| 8.580 \| **0.001** \| 0.277 \| 31.384 \| **0.001** \| \| 57.366 \| **0.001** \| \| 24.433 \| **0.001** \| \| O x E \| \| 0.103 \| 4.934 \| **0.001** \| 0.213 \| 11.672 \| **0.001** \| \| 8.141 \| **0.002** \| \| 5.109 \| **0.024** \| \|  \| O x G \| \| 0.083 \| 6.360 \| **0.001** \| 0.136 \| 11.052 \| **0.001** \| \| 17.564 \| **0.001** \| \| 14.545 \| **0.002** \| \|  \| O x I \| \| 0.128 \| 10.577 \| **0.001** \| 0.308 \| 31.996 \| **0.001** \| \| 129.54 \| **0.001** \| \| 46.591 \| **0.001** \| \|  \| O x S \| \| 0.040 \| 2.958 \| **0.001** \| 0.064 \| 4.887 \| **0.003** \| \| 2.360 \| 0.116 \| \| 3.081 \| 0.073 \| \|  \| G x I \| \| 0.195 \| 25.167 \| **0.001** \| 0.409 \| 71.830 \| **0.001** \| \| 103.48 \| **0.001** \| \| 66.048 \| **0.001** \| \|  \| G x S \| \| 0.111 \| 12.900 \| **0.001** \| 0.109 \| 12.596 \| **0.001** \| \| 6.358 \| **0.011** \| \| 1.437 \| 0.225 \| \|  \| G x E \| \| 0.109 \| 9.184 \| **0.001** \| 0.119 \| 10.158 \| **0.001** \| \| 0.655 \| 0.429 \| \| 0.078 \| 0.779 \| \|  \| I x S \| \| 0.193 \| 25.076 \| **0.001** \| 0.416 \| 74.667 \| **0.001** \| \| 133.64 \| **0.001** \| \| 70.127 \| **0.001** \| \|  \| I x E \| \| 0.155 \| 14.124 \| **0.001** \| 0.335 \| 38.857 \| **0.001** \| \| 69.693 \| **0.001** \| \| 32.740 \| **0.001** \| \|  \| S x E \| \| 0.030 \| 2.340 \| **0.002** \| 0.048 \| 3.851 \| **0.008** \| \| 1.367 \| 0.235 \| \| 0.349 \| 0.575 \| \| **Sex** \| M x F \| \| 0.029 \| 7.073 \| **0.001** \| 0.029 \| 6.886 \| **0.001** \| \| 6.266 \| **0.011** \| \| 0.516 \| 0.493 \| \| **Sex Shared Sites (body site interaction)** \| \| Sex  Body Site  Sex:Body Site \| 0.017  0.227  0.009 \| 3.556  23.305  0.908 \| **0.001**  **0.001**  0.561 \| 0.007  0.455  0.006 \| 2.096  65.453  0.831 \| 0.101  **0.001**  0.474 \| \| 0.611  84.811  36.333 \| 0.433  **0.001**  **0.001** \| \| 0.059  57.721  22.709 \| 0.813  **0.001**  **0.001** \| \| **Pregnancy Status** \| P x NP \| \| 0.016 \| 2.447 \| **0.006** \| 0.017 \| 2.548 \| **0.030** \| \| 8.845 \| **0.002** \| \| 4.443 \| **0.029** \| \| **Pregnancy**  **Stage (Male samples ONLY)** \| All Preg Stages \| \| 0.039 \| 1.956 \| **0.002** \| 0.033 \| 1.651 \| 0.071 \| \| 3.690 \| **0.015** \| \| 2.540 \| 0.075 \| \| NP x EP \| \| 0.028 \| 2.546 \| **0.003** \| 0.032 \| 2.919 \| **0.021** \| \| 10.150 \| **0.001** \| \| 6.159 \| **0.015** \| \| NP x MP \| \| 0.036 \| 2.296 \| **0.007** \| 0.030 \| 1.894 \| 0.098 \| \| 4.384 \| **0.036** \| \| 2.058 \| 0.168 \| \| NP x LP \| \| 0.070 \| 3.064 \| **0.001** \| 0.059 \| 2.555 \| **0.022** \| \| 3.536 \| 0.086 \| \| 1.339 \| 0.258 \| \| EP x MP \| \| 0.014 \| 1.496 \| 0.080 \| 0.012 \| 1.313 \| 0.218 \| \| 1.781 \| 0.185 \| \| 1.573 \| 0.211 \| \| EP x LP \| \| 0.018 \| 1.543 \| **0.054** \| 0.012 \| 1.046 \| 0.334 \| \| 1.553 \| 0.195 \| \| 2.089 \| 0.161 \| \| MP x LP \| \| 0.030 \| 1.784 \| **0.029** \| 0.011 \| 0.654 \| 0.637 \| \| 0.038 \| 0.841 \| \| 0.187 \| 0.678 \| \| **Brood pouch Sites**  **ONLY** \| P BP x NP BP \| \| 0.057 \| 1.746 \| **0.018** \| 0.125 \| 4.149 \| **0.002** \| \| 9.549 \| **0.006** \| \| 4.492 \| **0.038** \| \| BP x M S \| \| 0.075 \| 4.843 \| **0.001** \| 0.225 \| 17.385 \| **0.001** \| \| 29.858 \| **0.001** \| \| 10.886 \| **0.001** \| \| P BP x E \| \| 0.077 \| 4.001 \| **0.001** \| 0.237 \| 14.874 \| **0.001** \| \| 34.093 \| **0.001** \| \| 11.754 \| **0.002** \| \| NP BP x E \| \| 0.083 \| 2.615 \| **0.001** \| 0.058 \| 1.794 \| 0.085 \| \| 0.419 \| 0.579 \| \| 0.009 \| 0.927 \| \| P BP x O \| \| 0.178 \| 9.324 \| **0.001** \| 0.454 \| 35.789 \| **0.001** \| \| 80.015 \| **0.001** \| \| 37.036 \| **0.001** \| \| NP BP x O \| \| 0.160 \| 4.557 \| **0.001** \| 0.218 \| 6.674 \| **0.002** \| \| 15.510 \| **0.002** \| \| 23.587 \| **0.001** \| \| All Preg Stages \| \| 0.138 \| 1.438 \| **0.004** \| 0.212 \| 2.417 \| **0.009** \| \| 3.598 \| **0.024** \| \| 1.699 \| 0.201 \| \| NP x EP \| \| 0.104 \| 1.971 \| **0.004** \| 0.202 \| 4.290 \| **0.006** \| \| 8.686 \| **0.009** \| \| 3.403 \| 0.078 \| \| NP x MP \| \| 0.131 \| 1.814 \| **0.002** \| 0.227 \| 3.519 \| **0.014** \| \| 3.272 \| 0.092 \| \| 1.301 \| 0.324 \| \| NP x LP \| \| 0.188 \| 1.849 \| **0.008** \| 0.349 \| 4.282 \| **0.013** \| \| 13.211 \| **0.004** \| \| 4.261 \| 0.089 \| \| EP x MP \| \| 0.054 \| 1.095 \| 0.244 \| 0.070 \| 1.436 \| 0.133 \| \| 2.318 \| 0.151 \| \| 1.541 \| 0.241 \| \| EP x LP \| \| 0.073 \| 1.176 \| 0.171 \| 0.077 \| 1.249 \| 0.247 \| \| 0.169 \| 0.695 \| \| 0.314 \| 0.633 \| \| MP x LP \| \| 0.104 \| 1.163 \| 0.179 \| 0.113 \| 1.270 \| 0.238 \| \| 0.938 \| 0.383 \| \| 0.413 \| 0.600 \| \| **Supplemental Table 3.** PERMANOVA and PERMDISP test on Weighted UniFrac and Manhattan Distance (Beta Diversity) with 999 permutations. Holm’s adjusted p-values were used for PERMANOVA to control for multiple comparisons. Significant p-values (*P ≤ 0.05*) are bolded. Brood pouch = BP, Embryo = E, Gill = G, Intestines = I, Ovaries = O, Skin = S, Male = M, Female = F, Pregnant = P, Non-pregnant = NP, Early pregnancy = EP, Mid pregnancy = MP, Late pregnancy = LP. \| \| \| \| \| \| \| \| \| \| \| \| \| \| \| | | | | | | | |

| Body  site | Abundance* | Phylum | Class | Order | Family | Genus | Species |
| --- | --- | --- | --- | --- | --- | --- | --- |
| Brood  Pouch | 0.190 | Bacteroidota | Bacteroidia | Flavobacteriales_877923 | Flavobacteriaceae | Croceitalea | litorea |
|  | 0.095 | Bacteroidota | Bacteroidia | Flavobacteriales_877923 | Flavobacteriaceae | Spongiimicrobium | salis |
|  | 0.077 | Bacteroidota | Bacteroidia | Flavobacteriales_877923 | Flavobacteriaceae | Croceitalea | unclassified |
|  | 0.070 | Cyanobacteria | Cyanobacteriia | Cyanobacteriales | Coleofasciculaceae_23353 | SIO2C1 | sp010672925 |
|  | 0.057 | Proteobacteria | Alphaproteobacteria | Rhodobacterales | Rhodobacteraceae | unclassified | unclassified |
|  | 0.046 | Bacteroidota | Bacteroidia | Flavobacteriales_877923 | Flavobacteriaceae | unclassified | unclassified |
|  | 0.039 | Proteobacteria | Alphaproteobacteria | Rhodobacterales | Rhodobacteraceae | Sulfitobacter_E_490551 | unclassified |
|  | 0.030 | Proteobacteria | Alphaproteobacteria | Rhodobacterales | Rhodobacteraceae | Litoreibacter | unclassified |
|  | 0.019 | Bacteroidota | Bacteroidia | Flavobacteriales_877923 | Flavobacteriaceae | Spongiivirga | citrea |
|  | 0.016 | **Proteobacteria** | **Gammaproteobacteria** | **Burkholderiales_597441** | **Methylophilaceae** | **Methylotenera_A_557637** | **oryzisoli** |
|  | 0.014 | **Planctomycetota** | **UBA1135** | **UBA1135** | **GCA-002686595** | **GCA-2862085** | **unclassified** |
|  | 0.011 | Bacteroidota | Bacteroidia | Chitinophagales | Saprospiraceae | Portibacter | lacus |
|  | 0.010 | Proteobacteria | Alphaproteobacteria | Rhizobiales_A_501396 | Rhizobiaceae_A_499470 | unclassified | unclassified |
|  | 0.010 | Proteobacteria | Gammaproteobacteria | Arenicellales | Arenicellaceae | Arenicella | unclassified |
|  | 0.010 | **Proteobacteria** | **Alphaproteobacteria** | **Rhodobacterales** | **Rhodobacteraceae** | **Yoonia_491068** | **unclassified** |
|  | 0.008 | Planctomycetota | Planctomycetia | Planctomycetales | Planctomycetaceae | Fuerstia | unclassified |
|  | 0.008 | **Planctomycetota** | **UBA1135** | **UBA1135** | **GCA-002686595** | **Pla163** | **sp007750655** |
|  | 0.007 | Planctomycetota | Planctomycetia | Pirellulales | Pirellulaceae | Mariniblastus | sp011087765 |
|  | 0.007 | **Proteobacteria** | **Gammaproteobacteria** | **Burkholderiales_597441** | **Methylophilaceae** | **Methylotenera_A_557637** | **mobilis** |
|  | 0.007 | **Verrucomicrobiota** | **Verrucomicrobiae** | **Verrucomicrobiales** | **Akkermansiaceae** | **Roseibacillus_B** | **unclassified** |
| Embryo | 0.139 | Bacteroidota | Bacteroidia | Flavobacteriales_877923 | Flavobacteriaceae | Croceitalea | litorea |
|  | 0.130 | Cyanobacteria | Cyanobacteriia | Cyanobacteriales | Coleofasciculaceae_23353 | SIO2C1 | sp010672925 |
|  | 0.088 | Bacteroidota | Bacteroidia | Flavobacteriales_877923 | Flavobacteriaceae | Spongiimicrobium | salis |
|  | 0.064 | Bacteroidota | Bacteroidia | Flavobacteriales_877923 | Flavobacteriaceae | Croceitalea | unclassified |
|  | 0.034 | Proteobacteria | Alphaproteobacteria | Rhodobacterales | Rhodobacteraceae | Sulfitobacter_E_490551 | unclassified |
|  | 0.028 | Proteobacteria | Alphaproteobacteria | Rhodobacterales | Rhodobacteraceae | unclassified | unclassified |
|  | 0.022 | Bacteroidota | Bacteroidia | Flavobacteriales_877923 | Flavobacteriaceae | unclassified | unclassified |
|  | 0.013 | Proteobacteria | Alphaproteobacteria | Rhodobacterales | Rhodobacteraceae | Litoreibacter | unclassified |
|  | 0.012 | Bacteroidota | Bacteroidia | Chitinophagales | Saprospiraceae | Portibacter | lacus |
|  | 0.012 | Bacteroidota | Bacteroidia | Flavobacteriales_877923 | Flavobacteriaceae | Spongiivirga | citrea |
|  | 0.012 | **Proteobacteria** | **Gammaproteobacteria** | **Enterobacterales_A_737866** | **Vibrionaceae** | **Vibrio_678715** | **unclassified** |
|  | 0.010 | Proteobacteria | Gammaproteobacteria | Arenicellales | Arenicellaceae | Arenicella | unclassified |
|  | 0.005 | Proteobacteria | Alphaproteobacteria | Rhizobiales_A_501396 | Rhizobiaceae_A_499470 | unclassified | unclassified |
| Gill | 0.296 | Proteobacteria | Alphaproteobacteria | Rhodobacterales | Rhodobacteraceae | unclassified | unclassified |
|  | 0.097 | **Proteobacteria** | **Alphaproteobacteria** | **Rhodobacterales** | **Rhodobacteraceae** | **Pikeienuella** | **unclassified** |
|  | 0.077 | Proteobacteria | Gammaproteobacteria | Burkholderiales_592524 | unclassified | unclassified | unclassified |
|  | 0.061 | **Proteobacteria** | **Alphaproteobacteria** | **Rhodobacterales** | **Rhodobacteraceae** | **Sulfitobacter_E_490551** | **brevis** |
|  | 0.041 | Bacteroidota | Bacteroidia | Flavobacteriales_877923 | Flavobacteriaceae | Croceitalea | litorea |
|  | 0.040 | **Proteobacteria** | **Gammaproteobacteria** | **Francisellales** | **Francisellaceae** | **Francisella_A** | **unclassified** |
|  | 0.038 | **Proteobacteria** | **Gammaproteobacteria** | **Burkholderiales_592524** | **Burkholderiaceae_A_580492** | **Polynucleobacter** | **unclassified** |
|  | 0.038 | **Proteobacteria** | **Alphaproteobacteria** | **Rickettsiales** | **unclassified** | **unclassified** | **unclassified** |
|  | 0.024 | **Cyanobacteria** | **Cyanobacteriia** | **PCC-6307** | **Cyanobiaceae** | **Vulcanococcus** | **sp000179255** |
|  | 0.015 | Cyanobacteria | Cyanobacteriia | Cyanobacteriales | Coleofasciculaceae_23353 | SIO2C1 | sp010672925 |
|  | 0.012 | **Bacteroidota** | **Bacteroidia** | **Flavobacteriales_877923** | **Flavobacteriaceae** | **Dokdonia** | **pacifica** |
|  | 0.009 | Bacteroidota | Bacteroidia | Flavobacteriales_877923 | Flavobacteriaceae | Patiriisocius | marinistellae |
|  | 0.007 | Bacteroidota | Bacteroidia | Flavobacteriales_877923 | Flavobacteriaceae | unclassified | unclassified |
|  | 0.006 | **Proteobacteria** | **Alphaproteobacteria** | **Rhodobacterales** | **Rhodobacteraceae** | **Planktomarina** | **temperata** |
|  | 0.005 | **Actinobacteriota** | **Actinomycetia** | **Sporichthyales** | **Sporichthyaceae** | **Longivirga** | **aurantiaca** |
| Intestines | 0.058 | Proteobacteria | Alphaproteobacteria | Rhodobacterales | Rhodobacteraceae | unclassified | unclassified |
|  | 0.055 | Cyanobacteria | Cyanobacteriia | Cyanobacteriales | Coleofasciculaceae_23353 | SIO2C1 | sp010672925 |
|  | 0.044 | **Desulfobacterota_I** | **Desulfobulbia** | **Desulfobulbales** | **Desulfocapsaceae** | **Desulfofustis** | **glycolicus** |
|  | 0.038 | **Proteobacteria** | **Gammaproteobacteria** | **Pseudomonadales_650612** | **Halieaceae** | **unclassified** | **unclassified** |
|  | 0.026 | **Bacteroidota** | **Bacteroidia** | **Flavobacteriales_877923** | **Flavobacteriaceae** | **Lutimonas** | **unclassified** |
|  | 0.026 | **Proteobacteria** | **Alphaproteobacteria** | **Rhodobacterales** | **Rhodobacteraceae** | **Rhodobacter_B_492605** | **unclassified** |
|  | 0.020 | Planctomycetota | Planctomycetia | Pirellulales | Pirellulaceae | Mariniblastus | sp011087765 |
|  | 0.020 | **Proteobacteria** | **Gammaproteobacteria** | **Chromatiales_602961** | **Sedimenticolaceae** | **GCF-000787395** | **unclassified** |
|  | 0.020 | **Planctomycetota** | **Planctomycetia** | **Pirellulales** | **Pirellulaceae** | **Rubripirellula** | **unclassified** |
|  | 0.016 | **Proteobacteria** | **Gammaproteobacteria** | **Methylococcales** | **Cycloclasticaceae** | **unclassified** | **unclassified** |
|  | 0.012 | Bacteroidota | Bacteroidia | Flavobacteriales_877923 | Flavobacteriaceae | Croceitalea | litorea |
|  | 0.012 | Planctomycetota | Planctomycetia | Planctomycetales | Planctomycetaceae | Fuerstia | unclassified |
|  | 0.012 | **Actinobacteriota** | **Acidimicrobiia_401430** | **Acidimicrobiales** | **Ilumatobacteraceae** | **Ilumatobacter_A** | **nonamiensis** |
|  | 0.011 | **Chloroflexota** | **Anaerolineae** | **Caldilineales** | **Caldilineaceae** | **unclassified** | **unclassified** |
|  | 0.011 | Proteobacteria | Alphaproteobacteria | Rhizobiales_A_501396 | Rhizobiaceae_A_499470 | unclassified | unclassified |
|  | 0.010 | **Desulfobacterota_I** | **Desulfobulbia** | **Desulfobulbales** | **Desulfocapsaceae** | **Desulforhopalus** | **unclassified** |
|  | 0.009 | Proteobacteria | Alphaproteobacteria | Rhodobacterales | Rhodobacteraceae | Sulfitobacter_E_490551 | unclassified |
|  | 0.009 | Bacteroidota | Bacteroidia | Flavobacteriales_877923 | Flavobacteriaceae | Flavobacterium | unclassified |
|  | 0.009 | **Proteobacteria** | **Gammaproteobacteria** | **DSM-26407** | **DSM-26407** | **Thioalbus** | **unclassified** |
|  | 0.008 | Proteobacteria | Gammaproteobacteria | unclassified | unclassified | unclassified | unclassified |
|  | 0.008 | **Proteobacteria** | **Gammaproteobacteria** | **DSM-19610** | **DSM-19610** | **Thiogranum** | **unclassified** |
| Ovaries | 0.217 | Bacteroidota | Bacteroidia | Flavobacteriales_877923 | Flavobacteriaceae | Croceitalea | litorea |
|  | 0.202 | Cyanobacteria | Cyanobacteriia | Cyanobacteriales | Coleofasciculaceae_23353 | SIO2C1 | sp010672925 |
|  | 0.028 | **Proteobacteria** | **Gammaproteobacteria** | **Enterobacterales_A_737866** | **Alteromonadaceae_665222** | **Alishewanella_662828** | **unclassified** |
|  | 0.027 | Bacteroidota | Bacteroidia | Flavobacteriales_877923 | Flavobacteriaceae | Flavobacterium | unclassified |
|  | 0.025 | Proteobacteria | Alphaproteobacteria | Rhodobacterales | Rhodobacteraceae | unclassified | unclassified |
|  | 0.020 | Proteobacteria | Gammaproteobacteria | Arenicellales | Arenicellaceae | Arenicella | unclassified |
|  | 0.020 | Bacteroidota | Bacteroidia | Flavobacteriales_877923 | Flavobacteriaceae | unclassified | unclassified |
|  | 0.019 | Bacteroidota | Bacteroidia | Flavobacteriales_877923 | Flavobacteriaceae | Spongiivirga | citrea |
|  | 0.017 | Firmicutes_D | Bacilli | Staphylococcales | Staphylococcaceae | Staphylococcus | unclassified |
|  | 0.011 | **Actinobacteriota** | **Actinomycetia** | **Mycobacteriales** | **Mycobacteriaceae** | **Corynebacterium** | **unclassified** |
|  | 0.010 | **Proteobacteria** | **Gammaproteobacteria** | **Pseudomonadales_660879** | **Moraxellaceae** | **Acinetobacter** | **unclassified** |
|  | 0.009 | **Proteobacteria** | **Gammaproteobacteria** | **Enterobacterales_A_737866** | **Alteromonadaceae_665222** | **Pararheinheimera** | **texasensis** |
|  | 0.009 | **Proteobacteria** | **Gammaproteobacteria** | **Burkholderiales_592522** | **Burkholderiaceae_A_592522** | **unclassified** | **unclassified** |
|  | 0.007 | **Firmicutes_D** | **Bacilli** | **Lactobacillales** | **Streptococcaceae** | **Streptococcus** | **unclassified** |
|  | 0.007 | Proteobacteria | Gammaproteobacteria | Burkholderiales_592524 | unclassified | unclassified | unclassified |
|  | 0.005 | Bacteroidota | Bacteroidia | Flavobacteriales_877923 | Flavobacteriaceae | Patiriisocius | marinistellae |
| Skin | 0.399 | Bacteroidota | Bacteroidia | Flavobacteriales_877923 | Flavobacteriaceae | Croceitalea | litorea |
|  | 0.124 | Cyanobacteria | Cyanobacteriia | Cyanobacteriales | Coleofasciculaceae_23353 | SIO2C1 | sp010672925 |
|  | 0.041 | Proteobacteria | Alphaproteobacteria | Rhodobacterales | Rhodobacteraceae | unclassified | unclassified |
|  | 0.030 | Bacteroidota | Bacteroidia | Flavobacteriales_877923 | Flavobacteriaceae | unclassified | unclassified |
|  | 0.027 | Proteobacteria | Gammaproteobacteria | Arenicellales | Arenicellaceae | Arenicella | unclassified |
|  | 0.026 | Bacteroidota | Bacteroidia | Flavobacteriales_877923 | Flavobacteriaceae | Spongiimicrobium | salis |
|  | 0.020 | Bacteroidota | Bacteroidia | Flavobacteriales_877923 | Flavobacteriaceae | Spongiivirga | citrea |
|  | 0.012 | Proteobacteria | Alphaproteobacteria | Rhodobacterales | Rhodobacteraceae | Litoreibacter | unclassified |
|  | 0.011 | Bacteroidota | Bacteroidia | Flavobacteriales_877923 | Flavobacteriaceae | Patiriisocius | marinistellae |
|  | 0.011 | Firmicutes_D | Bacilli | Staphylococcales | Staphylococcaceae | Staphylococcus | unclassified |
|  | 0.010 | Proteobacteria | Alphaproteobacteria | Rhodobacterales | Rhodobacteraceae | Sulfitobacter_E_490551 | unclassified |
|  | 0.006 | Proteobacteria | Alphaproteobacteria | Rhizobiales_A_501396 | Rhizobiaceae_A_499470 | unclassified | unclassified |
|  | 0.005 | Proteobacteria | Gammaproteobacteria | unclassified | unclassified | unclassified | unclassified |
|  | 0.005 | Bacteroidota | Bacteroidia | Flavobacteriales_877923 | Flavobacteriaceae | Flavobacterium | unclassified |
|  | 0.005 | Bacteroidota | Bacteroidia | Chitinophagales | Saprospiraceae | Portibacter | lacus |

**Supplementary Table 4**. Core microbiome of all body sites with ASVs classified to the lowest taxonomic resolution. Bolded taxa are the ASVs unique to a specific body site (i.e., not found in any other core body site). * The relative abundance is calculated as the proportion of each ASV in relation to the total microbial community within each specific body site sample.

**Supplementary Table 5**. Indicator species analysis results (attached as excel files to repository).


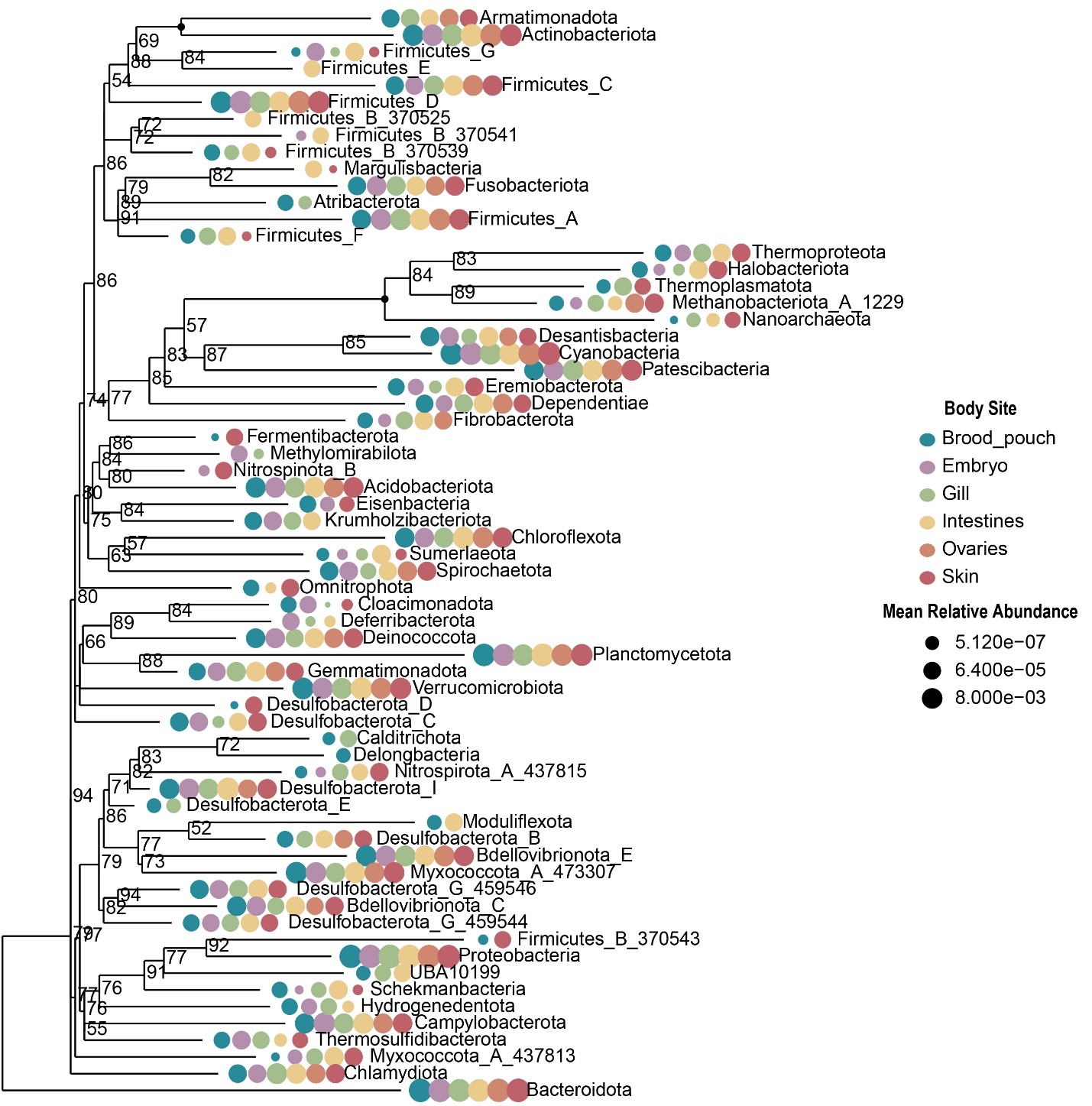


**Supplemental Figure 1**. Phylogenetic tree illustrating the 65 phyla within the bay pipefish microbiome, where colors denote different body sites and circle sizes represent the mean relative abundance at each site.


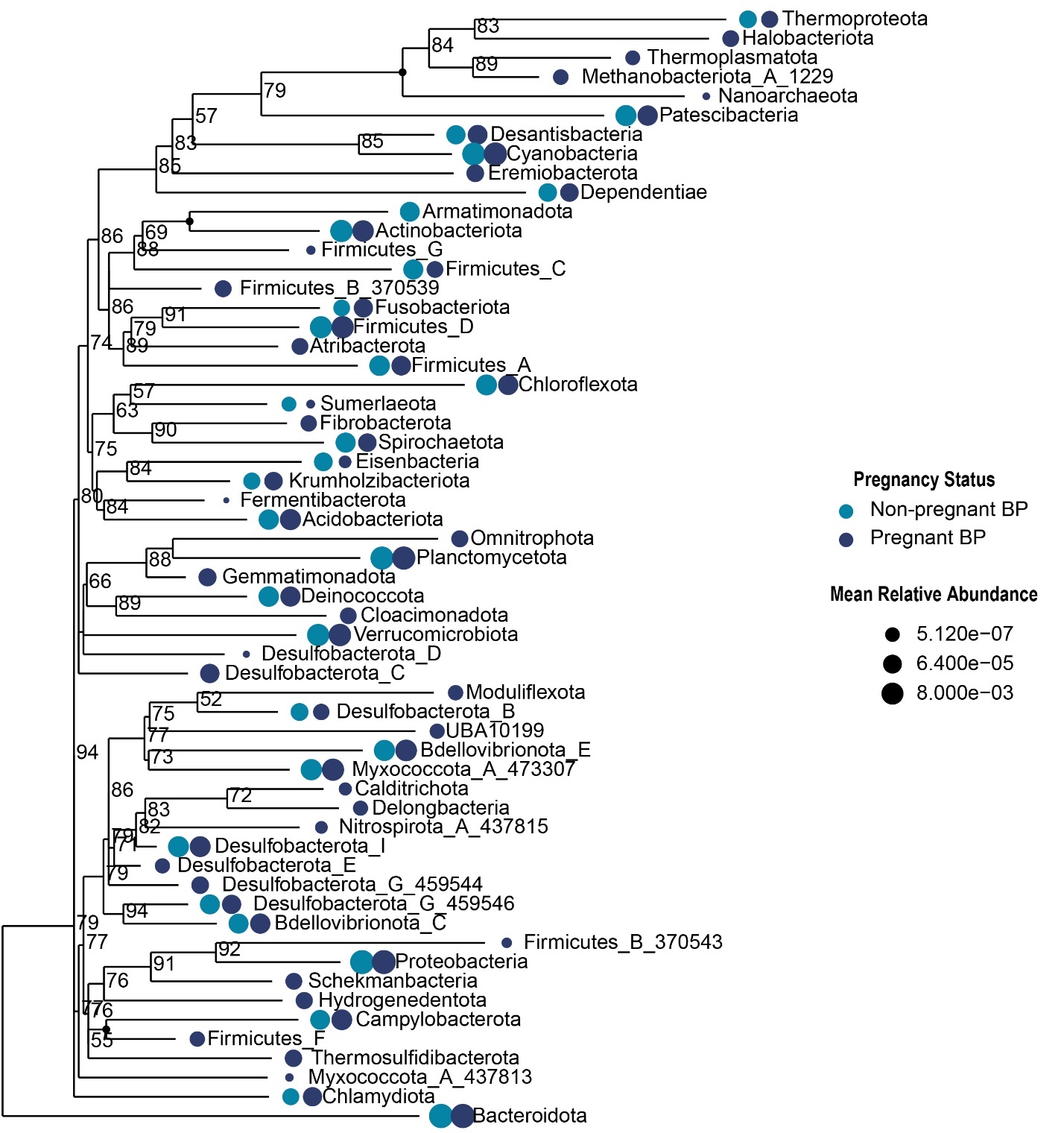


**Supplemental Figure 2**. Phylogenetic tree illustrating the 58 phyla present in the brood pouch microbiome by pregnancy status. Circle colors denote either pregnant or non-pregnant brood pouch samples and circle sizes represent the mean relative abundance at each site. BP = Brood pouch.


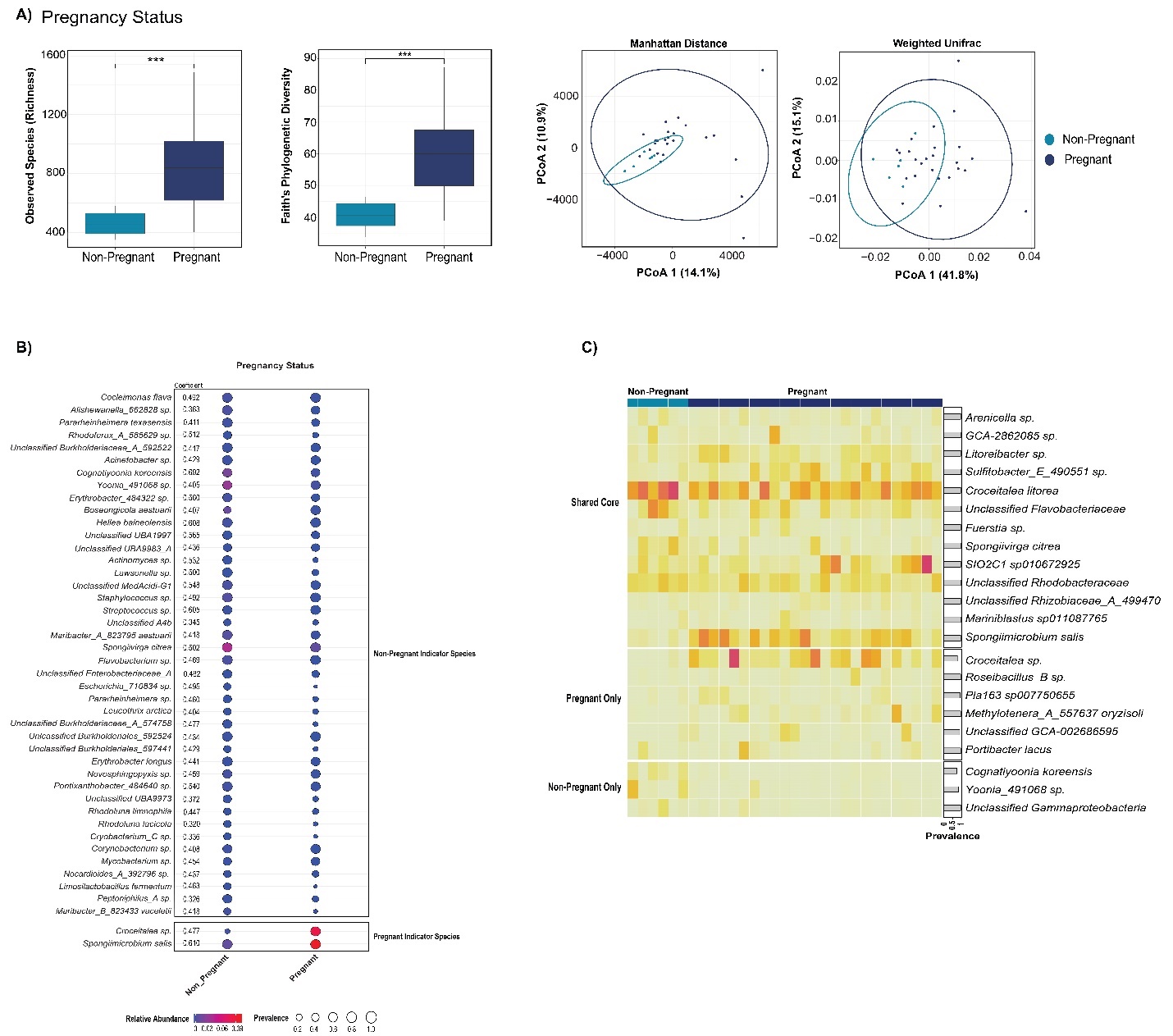


**Supplementary Figure 3.** (A) Richness, Faith’s PD (alpha diversity), Manhattan distance, and Weighted UniFrac metrics (beta diversity) of ASVs were analyzed for brood pouch samples by (A) pregnancy status (pregnant vs. non-pregnant). (B) Indicator species and (C) core microbiome analysis of pregnant and non-pregnant brood pouch samples.

**Supplementary Figure 4.** Indicator species analysis identifies ASVs significantly associated with pregnancy stage in brood pouch samples with their respective indicator coefficient (p ≤ 0.01). Species are grouped by pregnancy stage, with boxes displaying the selected indicators. Circle size represents prevalence, color indicates abundance, and indicator values (0–1) reflect site fidelity, with higher values indicating stronger associations.

**Supplementary Figure 5.** Alpha and beta diversity comparison between the sexes considering all body sites (brood pouch, embryos, ovaries, gill, intestines, outer skin) with an emphasis on male and female differences in shared body sites (gill, intestines, outer skin). The alpha diversity metrics are observed species richness (A), Shannon Index (B), inverse Simpson index (C), and Faith’s phylogenetic diversity (D). Two-dimensional principal coordinates analysis (PcoA) of microbial communities between males and females, using (E) Manhattan distance and (F) Weighted UniFrac metrics with 95% confidence ellipses. Statistically significant differences between groups are denoted using asterisk notation (p > 0.05 ns, p ≤ 0.05*, p ≤ 0.01**, p ≤ 0.001***, p ≤ 0.0001****).

**Supplementary Figure 6**. (A) Core microbiome and (B) Indicator species analysis identifies ASVs that are significantly associated with male or female samples, focusing only on the shared body sites (gills, intestines, and skin). (A) Heatmap of the core microbiome by sex and body site, organized by shared core species, followed by male-exclusive core, and then female-exclusive core. (B) Circle size represents prevalence, while color indicates abundance. The indicator values (coefficient) reflect site fidelity, with higher values showing a stronger association between the ASV and a specific sex. ASVs were only considered indicator species if p ≤ 0.001.

**Supplemental Results** **and Discussion**

***Core microbiomes and indicator species reflect taxonomic consistency and fidelity with changes in pregnancy status***

The most abundant phyla unique to pregnant brood pouches were Desulfobacterota_C (0.014%), Gemmatimonadota (0.003%), Eremiobacterota (0.001%), Desulfobacterota_G_459544 (0.001%), Thermosulfidibacterota (0.001%), and Hydrogenedentota (0.0008%). At the level of ASVs, pregnant and non-pregnant brood pouches shared 37.4% of all identified agglomerated ASVs (651 out of 1741 total ASVs found in the brood pouch sites). Non-pregnant brood pouches included a total of 742 agglomerated ASVs present with 91 unique taxa, while pregnant brood pouches represented 1650 ASVs present with 999 taxa exclusive to them when only considering pregnancy status. Among these unique taxa in pregnant brood pouches, the most abundant were *Uncultured Spiroplasma sp.* (1.4% mean relative abundance, 64% prevalence), *Kiloniella laminariae* (0.77% mean relative abundance, 48% prevalence), *Roseovarius nanhaiticus* (0.32% mean relative abundance, 44% prevalence), *Pontixanthobacter luteolus* (0.14% mean relative abundance, 68% prevalence), and *Pacificitalea manganoxidans* (0.12% mean relative abundance, 44% prevalence). Despite their lower mean relative abundances, *UBA8403 sp002325615* (0.09% mean relative abundance, 76% prevalence) and *Unclassified Chlamydiales_778124* (0.007% mean relative abundance, 76% prevalence) stood out as the most prevalent taxa unique to pregnant brood pouches. The most abundant taxa unique to the non-pregnant brood pouches included *Prevotella multiformis* (0.05% mean relative abundance), *Chthonomonas sp.* (0.03%), *Planktophila sp001438925* (0.02%), *Roseomonas_A_507160 sp.* (0.02%), and *Sulfuriflexus sp.* (0.02%) each having a prevalence of 17%. Meanwhile, *Unclassified Enterococcaceae* (0.02%), *Centipeda periodonti*i (0.01%), *UBA6984 sp003258725* (0.01%), *Paenarthrobacter sp.* (0.01%), and *UBA1573 sp001767915* (0.004%) stood out as the most prevalent taxa, each at 33% prevalence.

Each pregnancy status harbored some unique core ASVs that could indicate functional importance to those specific stages during embryo development. In non-pregnant brood pouches, three unique ASVs were identified: *Yoonia_491068 sp.* was the most abundant at 3.7% with 83% prevalence, followed by *Cognatiyoonia koreensis* at 2.3% abundance and 83% prevalence, and *Unclassified Gammaproteobacteria* at 1.3% abundance, present in all non-pregnant samples. Early pregnancy also featured three unique ASVs, with *Unclassified GCA-002686595* being the most abundant and prevalent (2% relative abundance and 92% prevalence). Mid pregnancy had four unique ASVs, with *Unclassified Methylophagaceae* being the most abundant at 1% and present in all samples.

***ANCOM-BC***
To assess if these ASVs considered indicator and/or core species maintain their abundance across body sites, we conducted a differential abundance analysis using ANCOM-BC. This was implemented to understand how these core/indicator species vary in relative abundance among body sites. The analysis revealed that nine ASVs were enriched (q ≤ 0.01) in the brood pouch relative to all other body sites: *Litoreibacter sp.*, *Sulfitobacter_E_490551 sp.*, *Unclassified Nannocystales*, *Henriciella sp001854405*, *Unclassified GCA-002705445*, *Methylotenera_A_557637 oryzisoli*, *Pla163 sp007750655*, *UBA11889 sp.*, and *Spongiimicrobium salis*. Conversely, we identified three ASV, *Unclassified Rhodobacteraceae*, *Unclassified Flavobacteriaceae*, and *SIO2C1 sp010672925*, that, despite being part of the core/indicator microbiome of the brood pouch, were depleted in the brood pouch relative to the other body sites. Stage-specific investigation identified six species enriched in non-pregnancy. *Actinomyces sp.* was depleted in all pregnancy stages, *Unclassified Burkholderiaceae_A_574758* and *Unclassified Burkholderiaceae_A_592522* were depleted in early pregnancy, and *Unclassified Rhodobacteraceae*, *Hellea balneolensis*, and *Spongiivirga citrea* were depleted in late pregnancy relative to non-pregnant brood pouches. The taxa *Haloferula harenae*, *Sulfitobacter_E_490551 sp.*, *Croceitalea sp.*, *Methylotenera_A_557637 oryzisoli*, *Roseibacillus_B sp.*, and *Litoreibacter sp.* were differentially abundant between non-pregnant and early pregnancy stages, with depletion in non-pregnancy and enrichment in early pregnancy. *Croceitalea sp.*, *Methylotenera_A_557637 oryzisoli*, and *Litoreibacter sp.* were enriched in the mid-pregnancy stage relative to the non-pregnant state. *Unclassified GCA-002705445* becomes differentially abundant in mid pregnancy relative to non-pregnancy. *Xenococcus sp000332055* was enriched specifically in late pregnancy and depleted in non-pregnancy. Four ASVs including *Pla163 sp007750655*, *Unclassified GCA-002705445*, *Spongiimicrobium salis*, *Methylotenera_A_557637 oryzisoli* were significantly enriched in males relative to females (q ≤ 0.01) and were all considered indicator species for males. Alternatively, ten ASVs which include *Hymenobacter sp007713685*, *Unclassified Tepidisphaeraceae*, *Hymenobacter terrae*, *WHSN01 sp*., *Portibacter sp*., *Succinivibrio sp000431835*, *Patiriisocius marinistellae*, *4572-13 sp002084585*, *Dokdonia pacifica*, and *Croceitalea litorea* were all significantly depleted in males relative to females (q ≤ 0.01). Although *Croceitalea litorea* is considered a core species for both sexes, its differential abundance is explained by differences in abundance alone, as ANCOM-BC does not account for prevalence. The core species analysis does consider prevalence, which can help explain why these taxa are identified as core for both males and females despite being differentially abundant in one sex versus the other.

***Known probiotics in the bay pipefish microbiome***

Probiotic bacteria are live microorganisms that support their host’s defense against pathogens by producing metabolites that prevent other microorganisms from colonizing or growing, or by competing with them for nutrients and space^1^. With traditional bacterial management strategies, such as chemicals and antibiotics, increasingly leading to antibiotic resistance and posing risks to hosts, researchers are now focusing on healthier alternatives, like probiotics, for disease treatment and prevention in aquaculture^2^. Studies have shown that supplementing larval fish with probiotics enhances their survival rates and resistance to pathogens^3^. In our research, we aimed to explore whether known marine probiotics are naturally present in the body sites of the bay pipefish, particularly in the brood pouch, embryos, and ovaries, to elucidate their potential contribute to a successful pregnancy by repelling harmful pathogens and supporting embryonic development. We reviewed the marine probiotic literature to identify published studies^4–7^ that reported known probiotic species. We compiled over 70 species from the review and searched our ASV table, identifying seven probiotic species present across various body sites. Although these known probiotic bacterial species were not considered part of any body site core microbiome due to their lower relative abundances and prevalence, they could still play an important role in the microbiome community. Both *Bifidobacterium bifidum* and *Lactobacillus delbrueckii* were detected in the ovaries (0.012% mean relative abundance, 5% prevalence), intestines (0.0004%, 2%), and gills (0.000008%, 2%). *Limosilactobacillus fermentum* was most abundant in the skin (0.002%), with decreasing levels in the intestines (0.0008%), gills (0.0005%), and brood pouch (0.0004%), but equally prevalent in the brood pouch and skin (13%), followed by the gills (12%) and intestines (6%). *Weizmannia coagulans*, *Roseobacter sp.*, and *Kocuria sp.* were present in all body sites. *Weizmannia coagulans* was most abundant in the ovaries (1.44%), followed by the skin (0.41%), gills (0.21%), embryos (0.032%), intestines (0.025%), and brood pouch (0.0004%). Despite the lowest abundance in the brood pouch, it had the second highest prevalence (42%) after the ovaries (55%), followed by embryos (40%), gills (37%), intestines (30%), and skin (26%). *Roseobacter sp*. was most abundant in the intestines (0.49%), with decreasing levels in the ovaries (0.077%), embryos (0.018%), brood pouch (0.018%), skin (0.009%), and gills (0.000006%), and most prevalent in the intestines (83%), followed by the brood pouch (48%), skin (17%), embryos (16%), ovaries (10%), and gills (2%). *Kocuria sp.* was most abundant in the intestines (0.17%), with decreasing levels in the ovaries (0.084%), skin (0.017%), embryos (0.006%), gills (0.004%), and brood pouch (0.002%). Although the brood pouch had the lowest abundance, it had the highest prevalence (48%), followed by the intestines (37%), skin (36%), gills (29%), ovaries (25%), and embryos (24%). These specific bacterial species have been found to confer beneficial effects on growth in other fish species, produce secondary metabolites that improve larval survival, and invoke antagonistic activity against other teleost pathogens^1,6,8,9^. Although these probiotic bacteria were present in some body site samples at high prevalences, their relative abundances were quite low and therefore not found to be a core or indicator species. The highly dynamic nature of the microbiome, coupled with the limitations of short read 16S rRNA sequencing, can result in more abundant taxa overshadowing less abundant ones, potentially obscuring the detection of 'conditionally rare' taxa that may temporarily increase in abundance under optimal conditions^10,11^. Despite their low abundances, these taxa could still play crucial roles in shaping microbial composition and driving specific host functions^12^. This could be the case for the probiotic bacteria found in the bay pipefish body sites, where they are at low abundances, but in some cases, are highly prevalent in a specific body site. The presence of these probiotic microbes in the reproductive organs, as well as the other immunity-linked body sites like the intestines, could indicate that the bay pipefish is recruiting and maintaining beneficial symbionts to support a healthy, protective environment for developing embryos as the larval stage is the most susceptible to disease^13^.

**Supplemental References**

1. Vine, N. G., Leukes, W. D. & Kaiser, H. Probiotics in marine larviculture. *FEMS Microbiol Rev* **30**, 404–427 (2006).

2. Tarnecki, A. M., Wafapoor, M., Phillips, R. N. & Rhody, N. R. Benefits of a *Bacillus*  probiotic to larval fish survival and transport stress resistance. *Sci Rep* **9**, 4892 (2019).

3. Gatesoupe, F. J. The use of probiotics in aquaculture. *Aquaculture* **180**, 147–165 (1999).

4. Hoseinifar, S. H., Sun, Y.-Z., Wang, A. & Zhou, Z. Probiotics as Means of Diseases Control in Aquaculture, a Review of Current Knowledge and Future Perspectives. *Front. Microbiol.* **9**, 2429 (2018).

5. Liu, Y.-Y., Hsu, C.-Y., Yang, Y.-C., Huang, C.-H. & Chen, C.-C. ProbioMinServer: an integrated platform for assessing the safety and functional properties of potential probiotic strains. *Bioinformatics Advances* **3**, vbad153 (2023).

6. Probiotics and Prebiotics, Boosting Fish Gut Health, Immunity and Disease Resistance. *Int J Vet Sci* 212–223 (2024) doi:10.47278/book.CAM/2024.313.

7. Ibrahem, M. D. Evolution of probiotics in aquatic world: Potential effects, the current status in Egypt and recent prospectives. *Journal of Advanced Research* **6**, 765–791 (2015).

8. Fijan, S. Microorganisms with Claimed Probiotic Properties: An Overview of Recent Literature. *IJERPH* **11**, 4745–4767 (2014).

9. Hjelm, M., Riaza, A., Formoso, F., Melchiorsen, J. & Gram, L. Seasonal Incidence of Autochthonous Antagonistic *Roseobacter* spp. and *Vibrionaceae* Strains in a Turbot Larva ( *Scophthalmus maximus* ) Rearing System. *Appl Environ Microbiol* **70**, 7288–7294 (2004).

10. Saw, J. H. W. Characterizing the Uncultivated Microbial Minority: towards Understanding the Roles of the Rare Biosphere in Microbial Communities. *mSystems* **6**, 10.1128/msystems.00773-21 (2021).

11. Shade, A. *et al.* Conditionally Rare Taxa Disproportionately Contribute to Temporal Changes in Microbial Diversity. *mBio* **5**, e01371-14 (2014).

12. Han, G. & Vaishnava, S. Microbial underdogs: exploring the significance of low-abundance commensals in host-microbe interactions. *Exp Mol Med* **55**, 2498–2507 (2023).

13. Borges, N. *et al.* Bacteriome Structure, Function, and Probiotics in Fish Larviculture: The Good, the Bad, and the Gaps. *Annu. Rev. Anim. Biosci.* **9**, 423–452 (2021).
